# Nitrogen source type modulates heat stress response in coral symbiont (*Cladocopium goreaui*)

**DOI:** 10.1128/aem.00591-24

**Published:** 2025-01-07

**Authors:** Yulin Huang, Jiamin He, Yujie Wang, Ling Li, Senjie Lin

**Affiliations:** 1 State Key Laboratory of Marine Environmental Science, College of Ocean and Earth Sciences, Xiamen University 534813 , Xiamen, Fujian, China; 2 Department of Marine Sciences, University of Connecticut 197270 , Groton, Connecticut, USA; Unversidad de los Andes, Bogotá, Colombia

**Keywords:** coral reefs, symbiotic microalgae, nitrogen pollution, heat stress, transcriptome

## Abstract

Ocean warming due to climate change endangers coral reefs, and regional nitrogen overloading exacerbates the vulnerability of reef-building corals as the dual stress disrupts coral–Symbiodiniaceae mutualism. Different forms of nitrogen may create different interactive effects with thermal stress, but the underlying mechanisms remain elusive. To address the gap, we measured and compared the physiological and transcriptional responses of the Symbiodiniaceae *Cladocopium goreaui* to heat stress (31°C) when supplied with different types of nitrogen (nitrate, ammonium, or urea). Under heat stress (HS), cell proliferation and photosynthesis of *C. goreaui* declined, while cell size, lipid storage, and total antioxidant capacity increased, both to varied extents depending on the nitrogen type. Nitrate-cultured cells exhibited the most robust acclimation to HS, as evidenced by the fewest differentially expressed genes (DEGs) and less ROS accumulation, possibly due to activated nitrate reduction and enhanced ascorbate biogenesis. Ammonium-grown cultures exhibited higher algal proliferation and ROS scavenging capacity due to enhanced carotenoid and ascorbate quenching, but potentially reduced host recognizability due to the downregulation of N-glycan biosynthesis genes. Urea utilization led to the greatest ROS accumulation as genes involved in photorespiration, plant respiratory burst oxidase (RBOH), and protein refolding were markedly upregulated, but the greatest cutdown in photosynthate potentially available to corals as evidenced by photoinhibition and selfish lipid storage, indicating detrimental effects of urea overloading. The differential warming nitrogen-type interactive effects documented here has significant implication in coral–Symbiodiniaceae mutualism, which requires further research.

**IMPORTANCE:**

Regional nitrogen pollution exacerbates coral vulnerability to globally rising sea-surface temperature, with different nitrogen types exerting different interactive effects. How this occurs is poorly understood and understudied. This study explored the underlying mechanism by comparing physiological and transcriptional responses of a coral symbiont to heat stress under different nitrogen supplies (nitrate, ammonium, and urea). The results showed some common, significant responses to heat stress as well as some unique, N-source dependent responses. These findings underscore that nitrogen eutrophication is not all the same, the form of nitrogen pollution should be considered in coral conservation, and special attention should be given to urea pollution.

## INTRODUCTION

The coral reef ecosystem is a crucial marine ecosystem, providing food and shelter for almost a quarter of marine species and an estimated $2.7 trillion per year in goods and services (1). The mutualism between corals and dinoflagellate Symbiodiniaceae is the foundation of their ecological success, with corals providing shelter and nutrients to their endosymbiotic algae and receiving algal photosynthates in exchange (2–5). However, the symbiotic relationship is susceptible to environmental disturbances. Abnormal sea temperature rises due to global climate change disrupts the mutualism, leading to algal symbiont expulsion and coral bleaching (6, 7).

Regional or local water eutrophication adds another stress to corals. It has been widely documented that eutrophication, especially overloading of dissolved inorganic nitrogen (DIN), greatly increased the sensitivity of corals to thermal stress, with additive or synergistic effects (8–14). Oxidative stress in both partners is widely accepted as the major trigger of coral bleaching (15), and mounting evidence also suggests that the altered nitrogen (N) recycling between the partners might play a vital role in the onset of bleaching (e.g., 16–18 and references therein). Photosynthates of the algal symbionts are apportioned between algal proliferation and translocation to the coral host. Corals maximize carbon translocation by controlling nitrogen supply to the algae (16, 19–21). However, environmental N eutrophication can fuel algal proliferation, resulting in reduced photosynthate translocation, changing the role of the symbionts from mutualism to parasitism (17, 22, 23). In addition, thermal stress can stimulate host amino acid catabolism, providing ammonium to the algal symbionts (18, 24).

Furthermore, recent studies revealed that the nitrogen-heat stress interactive impacts on corals depend on the chemical form of the nitrogen (N) supply (13, 25–27). Nitrate, ammonium, and urea were three dominant N sources in coral reef waters (28). They can be readily assimilated by free-living (29) and coral-associated Symbiodiniaceae (30–35). Nitrate enrichment has been shown to reduce coral calcification, primary productivity, and carbon translocation, negative effects not observed for ammonium (8, 25, 26). Under heat stress, nitrate enrichment increased ROS and NO production in coral tissues, caused more extensive coral bleaching, and prolonged bleaching duration compared with urea or ammonium enrichment (13, 27). Isolated Symbiodiniaceae cultures, such as *Fugacium kawagutii* and *Effrenium voratum*, have been shown to prefer ammonium over nitrate (36, 37), consistent with the tenet that ammonium is preferable over nitrate because it can be assimilated directly into amino acids, whereas nitrate requires the energy-costing reduction to ammonium. However, the opposite has been found in some diatoms, which prefer nitrate (38–40), for unclear reasons.

To better understand how the chemical species of N interact with heat stress to impact coral health, it is important to first address how *ex hospite* Symbiodiniaceae responds to these combined factors. In the present study, a thermally sensitive Symbiodiniaceae that is widely distributed in the Pacific Ocean, *Cladocopium goreaui*, was chosen as the model for the investigation of this question. Physiological, biochemical, and transcriptomic analyses were integrated to examine the N type-heat stress interactive effects and shed light on the underlying mechanisms.

## RESULTS

### Cell population growth and cell size

**Fig 1:**
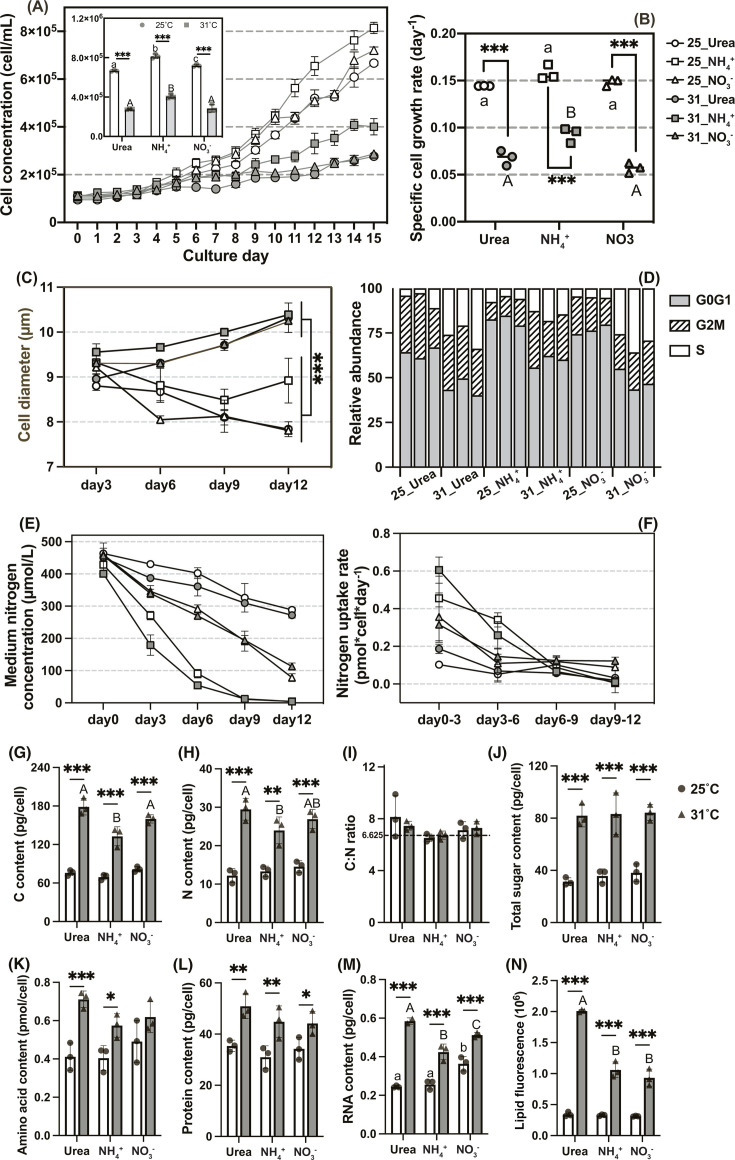
Heat stress responses of cell population growth, cell cycle, cell size, and cellular biochemical composition of *C. goreaui*. (**A**) Growth curve. (**B**) Specific cell growth rate. (**C**) Cell diameter. (**D**) Relative abundances of cells in different cell cycle phases (%). (**E**) Temporal change of media nitrogen concentrations. (**F**) Nitrogen uptake rates. (**G-I**) Cellular carbon, nitrogen contents, and C:N ratio. (**J-N**) Cellular sugar, amino acid, protein, RNA, and lipid contents. The inset in panel **A** shows the maximum cell concentration throughout the experimental period. Asterisks indicate statistically significant differences (*P<0.05, **P<0.01, ***P < 0.001) between two temperature treatments within the same N group. Different letters indicate significant differences between nutrient treatments under the same temperature condition. The data are mean ± SD (*n* = 3).

With roughly the same initial cell concentrations around 1×10^5^ cells/mL, growth curves of *Cladocopium goreaui* under different temperature conditions started to diverge since the sixth day of cultivation (MANOVA, *P* < 0.001). The exponential growth rate and maximum cell concentration at 25°C were roughly 2.05 and 2.25 times that at 31°C (two-way ANOVA, *P* < 0.001), indicating inhibitory effects of heat stress (HS). The NH_4_^+^ group (cultures containing ammonium chloride) grew more rapidly (RMANOVA, *P* < 0.01) and reached a higher maximum cell concentration (two-way ANOVA, *P* < 0.01) than the other two N groups (urea and NO_3_^-^) under both 25°C and 31°C (Fig. 1A and B).

Cell diameters of *C. goreaui* increased at 31°C but decreased at 25°C over time, with differences becoming significant since day 6 (MANOVA, *P* ≤ 0.001). Meanwhile, the 31°C cultures, regardless of N type, exhibited significantly elevated S-phase cell proportions and decreased G1-phase cell proportions, a symptom of cell cycle arrest in the S phase. The NH_4_^+^ group cells additionally exhibited an increase in the G2 and M phase cell proportions (two-way ANOVA, *P* < 0.01) (Fig. 1C and D).

### Nitrogen uptake and cellular biochemical composition

High temperature had a significant effect on N depletion in the medium (RMANOVA, *P* = 0.001), not on N uptake rates estimated on per cell basis (*P* = 0.132). In contrast, N type showed significant effects on both depletion and uptake rates (RMANOVA, *P* < 0.001), with ammonium > nitrate > urea (Fig. 1E and F). Cellular carbon and nitrogen contents both increased significantly under HS across all N-type culture groups (two-way ANOVA, *P* < 0.01). Cellular contents of sugar, amino acids, protein, neutral lipid, and RNA also increased significantly (two-way ANOVA, *P* < 0.05 except amino acid content in the NO_3_^-^ group). Under HS, urea-grown cells showed higher contents of carbon, lipid, and RNA than cells grown in nitrate or ammonium (two-way ANOVA, *P* < 0.05). The cellular C:N ratios stabilized at around the Redfield ratio (106:16) on the 12th day of the cultivation and were not affected by temperature or N types (Fig. 1G through N).

### Photosynthesis parameters, pigments, and Rubisco and D1 proteins

**Fig 2:**
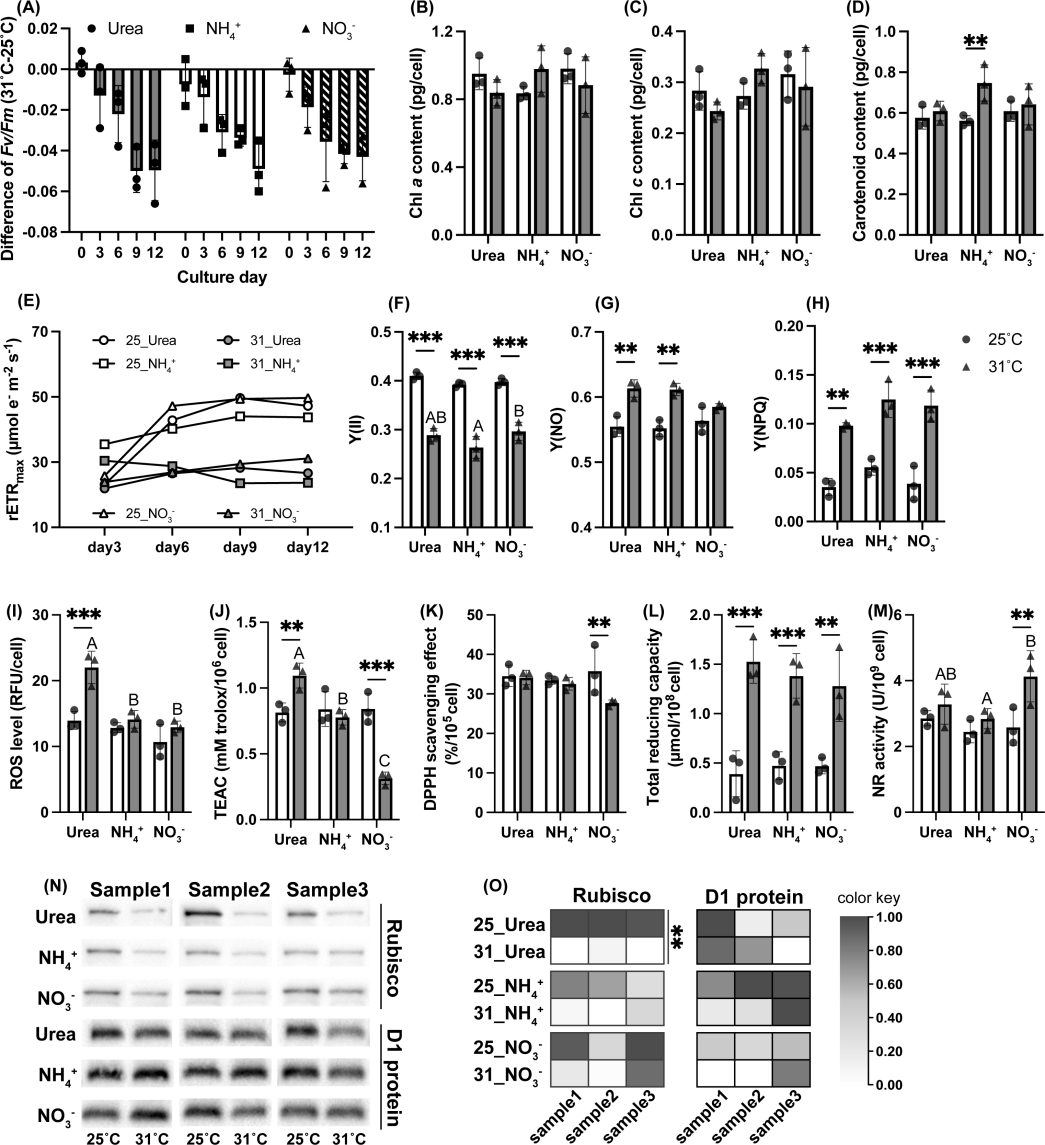
Heat stress responses of photosynthesis parameters, ROS, and anti-oxidant capacity in *C. goreaui*. (**A**) Temporal trends of differences in the *Fv/Fm* ratio between 31°C and 25°C, (*Fv/Fm*)_31_- (*Fv/Fm*)_25_. (**B-D**) Cellular chlorophyll *a*, chlorophyll *c*, and carotenoid contents. (**E**) Temporal trends of maximal relative electron transport rates. (**F-H**) Effective PSII photochemical efficiency, quantum yield of PSII non-regulated non-photochemical energy loss and quantum yield of PSII regulated non-photochemical energy loss. (**I**) Cellular ROS level. (**J-L**) Antioxidant capacity measured by different assays (ΑΒΤS, DPPH, and FRAP assays). (**M**) Nitrate reductase activity. (**N-O**) Western blotting of Rubisco and D1 protein of *C. goreaui* cells and the derived heatmap. Asterisks indicate significant statistical differences between two temperature treatments within the same nitrogen group (*P < 0.05, **P < 0.01, ***P < 0.001). Different letters indicate significant differences between nutrient treatments under the same temperature condition. The data in the bar charts are mean ± SD (*n* = 3). Original Western blot photos are provided in Fig. S5.

The photosystem II maximum quantum yield (*Fv/Fm*) decreased under HS in all N groups (RMANOVA, *P* < 0.001). The differences between 25°C and 31°C within each N group became significant since day 6 of the cultivation (MANOVA, *P* < 0.01). HS led to significant repression of PSII effective quantum yield Y(II) and elevation of regulated and non-regulated non-photochemical energy loss of PSII (Y(NO) and Y(NPQ), respectively) in each N group (two-way ANOVA, *P* < 0.05 except Y(NO) in the NO_3_^-^ group). Relative electron transfer rates (rETR) were consistently lower at 31°C (two-way ANOVA, *P* < 0.001). Cellular chlorophyll *a* and *c* contents under HS were lower in the Urea and NO_3_^-^ groups but higher in the NH_4_^+^ group, but all without statistical significance. However, ammonium-grown cells showed markedly increased cellular carotenoids under HS (two-way ANOVA, *P* = 0.005) (Figure 2A through H). By Western blotting, we found significant decreases in ribulose-1,5-bisphosphate carboxylase/oxygenase (Rubisco) protein abundance (in reference to total protein contents) under HS in the Urea group (two-way ANOVA, *P* = 0.006), but not in the NH_4_^+^ or NO_3_^-^ groups. Photosystem D1 protein was stable in heat-stressed *C. goreaui* regardless of N type (Fig. 2N and O).

### ROS and anti-oxidative capacity

Heat stress increased the cellular ROS contents of *C. goreaui* (two-way ANOVA, *P* < 0.001), but with statistical significance only for the Urea group (*P* < 0.001). Under HS, *C. goreaui* exhibited higher ABTS·^+^ scavenging efficiency when when grown on urea (*P* = 0.003), but indistinguishable and lower ABTS·^+^ and DPPH nitrogen radical scavenging when grown on ammonium and nitrate, respectively. Total reducing power increased under HS in all N groups (*P* < 0.01). HS also caused increases in nitrate reductase (NR) activity (two-way ANOVA, *P* = 0.006), but only the elevation in the nitrate-supplied group was significant (*P* = 0.003) (Fig. 2I through M).

### General transcriptional responses

**Fig 3:**
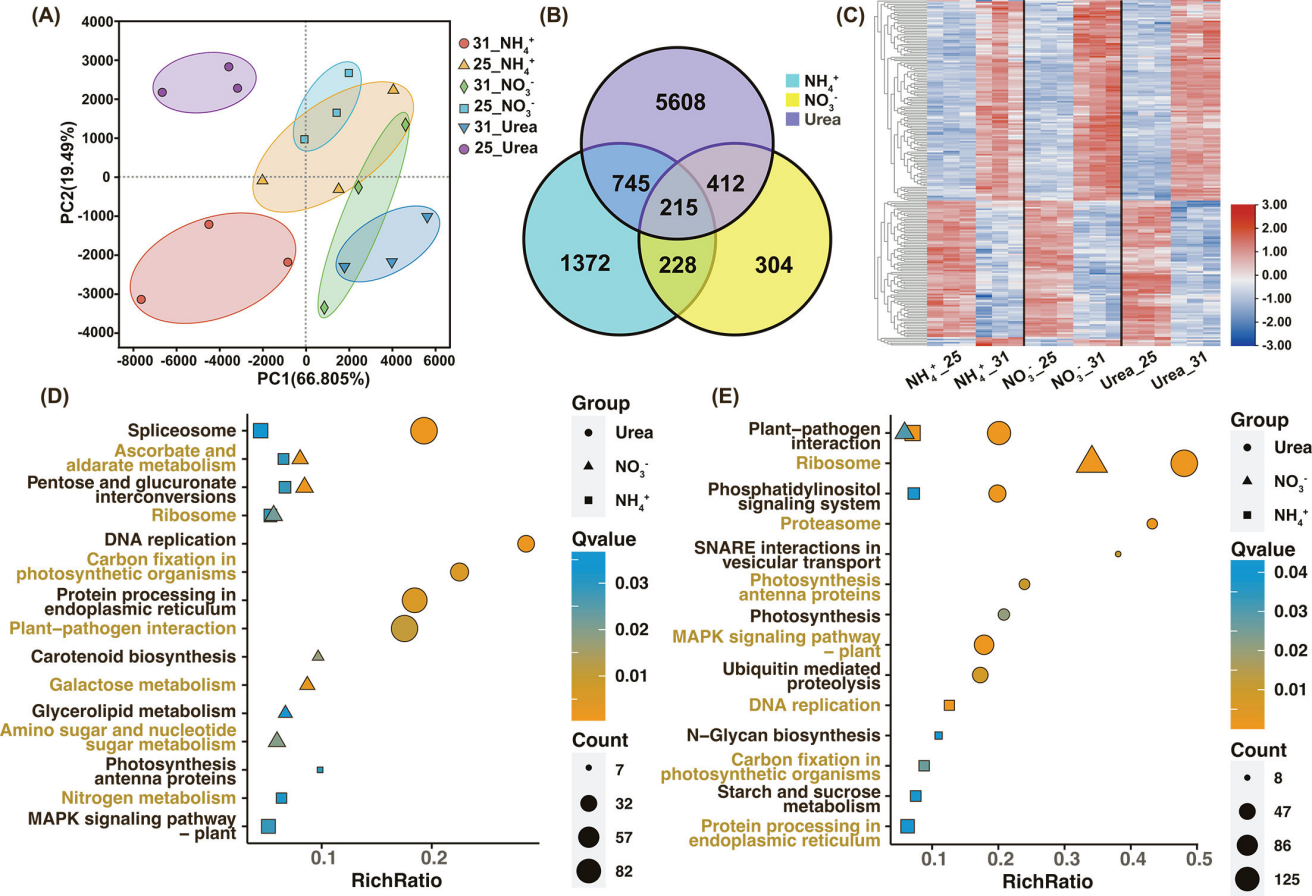
Transcriptomic responses to HS in *C. goreaui*. (**A**) Principal component analysis of transcriptomic data. (**B**) Venn diagram of HS-DEGs in the three different nutrient groups. (**C**) Heatmap of 215 HS-DEGs shared among the three nutrient groups. (**D-E**) KEGG enrichment of significantly HS-upregulated genes (D) and HS-downregulated genes (E).

An average of 43 million clean reads were yielded from each of the 18 RNA-seq samples. All clean reads data sets showed Q-20 values (error probability of 1%) >97.75% and total mapping ratios > 73.84% (Table S8). Principal component analysis (PCA) separated transcriptomic data obtained from 25°C and 31°C groups. Heat stress elicited differential expression of 2,560, 1,159, and 6,980 genes (defined as HS-DEGs) with cutoffs of fold-change (FC) > 1.5 in NH_4_^+^, NO_3_^-^, and Urea groups, respectively, accounting for 7%~19% of the total expressed genes (Table S8). This fold-change cutoff value was used due to small HS-DEG number when using FC > 2 (Fig. S1), consistent with previous frequent reports of low transcriptional regulation in dinoflagellates (41). KEGG enrichment plots of HS-DEGs indicated dissimilar transcriptomic HS responses of *C. goreaui* grown on different N (Fig. 3D and E). Among these HS-DEGs, 215 were shared by all three N groups, 211 of which showed concordant expression trends under HS, which are defined here as coHS-DEGs (Fig. 3A through C). According to GO annotation, the 211 coHS-DEGs were functionally related to inhibited cell division and pre-ribosome maturation, and upregulated phosphatidylinositol signaling (Fig. S2).

### Transcriptional response of the cell division cycle

Cyclins and cyclin-dependent kinases (CDKs) exhibited differential responses to heat stress under different N supply. Among the 12 cyclins detected in the transcriptomes, the Urea group showed three significantly downregulated cyclin genes (1 HS-DEG), and NH_4_^+^ and NO_3_^-^ groups both showed one significantly downregulated cyclin gene. For cell cycle-related CDKs (42), one CDK10 and three CDKA genes were upregulated, while two CDKA genes were downregulated in the Urea group, two CDKA were downregulated in the NH_4_^+^ group, and no CDK was regulated in the NO_3_^-^ group. CDK12/13, which is involved in transcription regulation and DNA damage response (43), was upregulated under HS regardless of N type. The Urea group additionally showed numerous upregulated genes related to DNA replication, pyrimidine and purin metabolism, and DNA damage response genes, such as DNA excision repair protein ERCC-2 and ERCC-3, and UV excision repair protein RAD23 (44). Urea-grown cells also exhibited more signals of cell cycle inhibition, as WEE1-like protein (WEE1) and cell division cycle 20 (CDC20) were upregulated (Table S7).

### Transcriptional response of N assimilation

**Fig 4:**
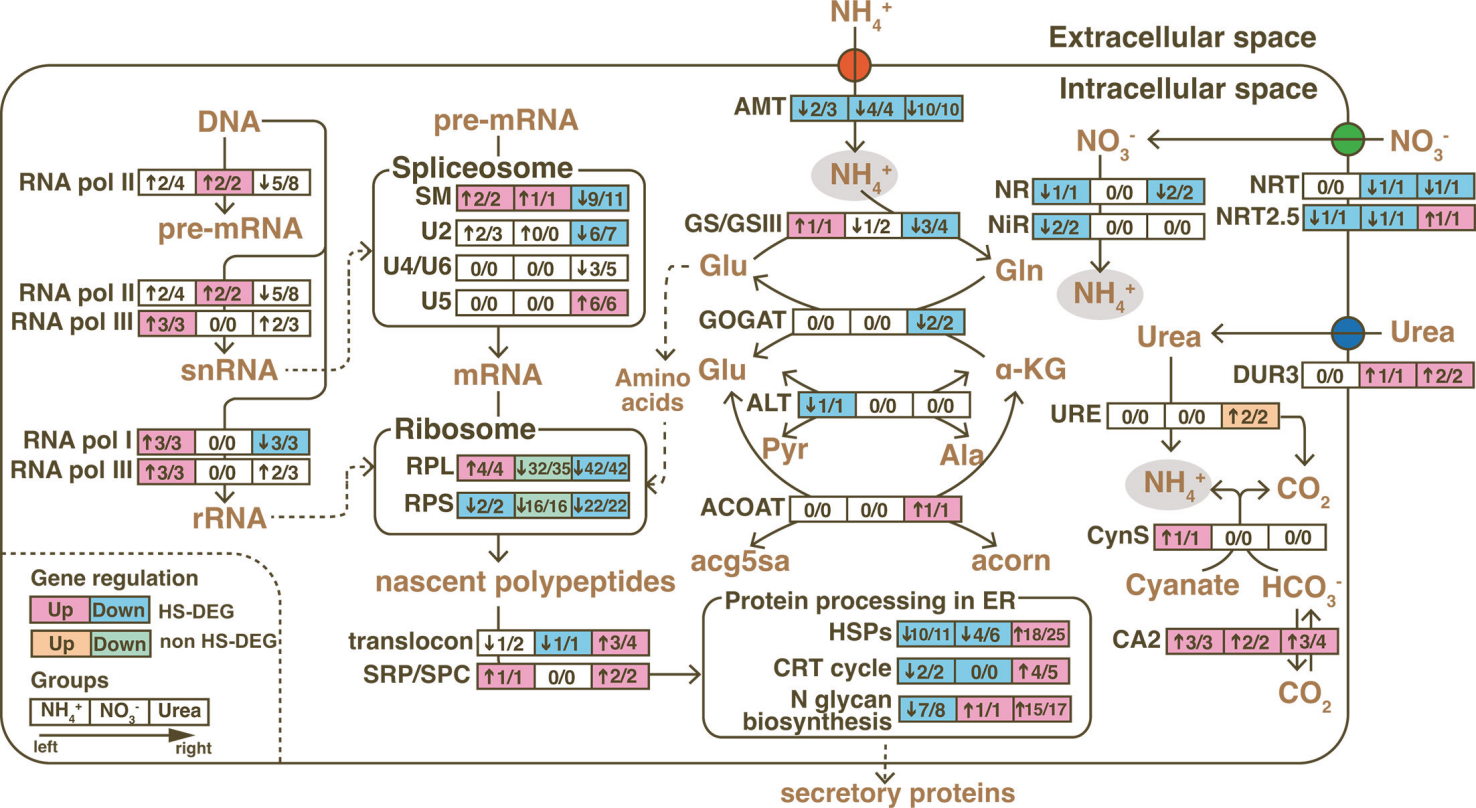
Schematic of transcriptional responses of N assimilation and protein biosynthesis to HS in *C. goreaui*. The numbers inside rectangles, from left to right, represent the count of up- or downregulated genes (indicated by arrows preceding the number)/the total number of regulated genes in the NH_4_^+^, NO_3_^-^or Urea group. Rectangles filled with pink or orange color indicate HS-upregulated genes, while blue or green color indicates HS-downregulated genes. Pink and blue colors signify numbers calculated from HS-DEGs, while the orange and green colors represent numbers calculated from significantly regulated genes (Q < 0.05 but with 2/3 ≤ FC(31°C/25°C) ≤3/2). Detailed gene descriptions can be found in Table S3.

As the transcriptome data showed, HS influenced the expression of nitrogen assimilation-related genes in *C. goreaui*. Across all N-type culture conditions, ammonium and nitrate transporters (AMT, NRT, and NRT2.5) were generally downregulated under HS. Urea proton symporter (DUR3) was upregulated by HS in the NO_3_^-^ and Urea groups. Under HS, nitrate reductase (NR) was downregulated in the NH_4_^+^ and Urea groups, and nitrite reductase (NiR) transcripts decreased only in the NH_4_^+^ group. Most genes related to ammonium assimilation process were downregulated under HS in the Urea group, including one glutamine synthetase (GS), two type III-glutamine synthetase (GSIII), and two NAD(P)H-dependent glutamine oxoglutarate aminotransferase or glutamate synthase (GOGAT-NADPH), indicating a potential decrease in urea-N assimilation into amino acids under HS. In contrast, no clear changes were seen in the GS/GOGAT genes in the NH_4_^+^ or NO_3_^-^ groups (Fig. 4).

### Transcriptional response of protein biosynthesis and processing

HS impacted the expression of genes involved in protein biosynthesis, with differences among N groups. In the Urea group, HS downregulated RNA polymerase I subunit genes, which transcribed rRNA, and most ribosomal protein genes (42 large subunit genes and 22 small subunit genes). Nitrate-grown cells also showed HS-induced downregulation of ribosomal proteins but with FC <1.5. Conversely, in the NH_4_^+^ group, RNA polymerase I and III were upregulated, alongside ribosomal large subunit genes, albeit with relatively few DEGs. While the ribosome transcription was limited, the HS-exposed Urea group showed significant upregulation of protein processing and maturating genes, including heat shock proteins (HSPs) and ER protein processing enzymes. However, most HSPs and protein-folding enzymes were downregulated in the NH_4_^+^ and NO_3_^-^ groups. Enzymes functioning in early N-glycan biosynthesis (N-glycan precursor biosynthesis and trimming) were upregulated in the Urea group but downregulated in the NH_4_^+^ group. Only one alpha-1,2-mannosyltransferase (ALG11) that catalyzed N-glycan precursor biosynthesis was upregulated in the NO_3_^-^ group (Fig. 4).

### Transcriptional responses of photosynthesis, energy metabolism, and lipid metabolism

In the NH_4_^+^ group, HS-DEGs encoded PSII and PSI components, photosynthesis antenna proteins, chlorophyll biosynthesis enzymes, and chloroplast-located FBA II were upregulated. In the NO_3_^-^ group, two HS-DEGs related to chlorophyll biosynthesis and the Calvin cycle were upregulated. However, in the Urea group, genes encoding photosystem components (PSII proteins, cytochrome b6f, ATP synthase), antenna proteins, and Calvin cycle enzymes (including 5 Rubisco genes) were downregulated under HS, but photorespiration-related genes were upregulated (Table S4).

**Fig 5:**
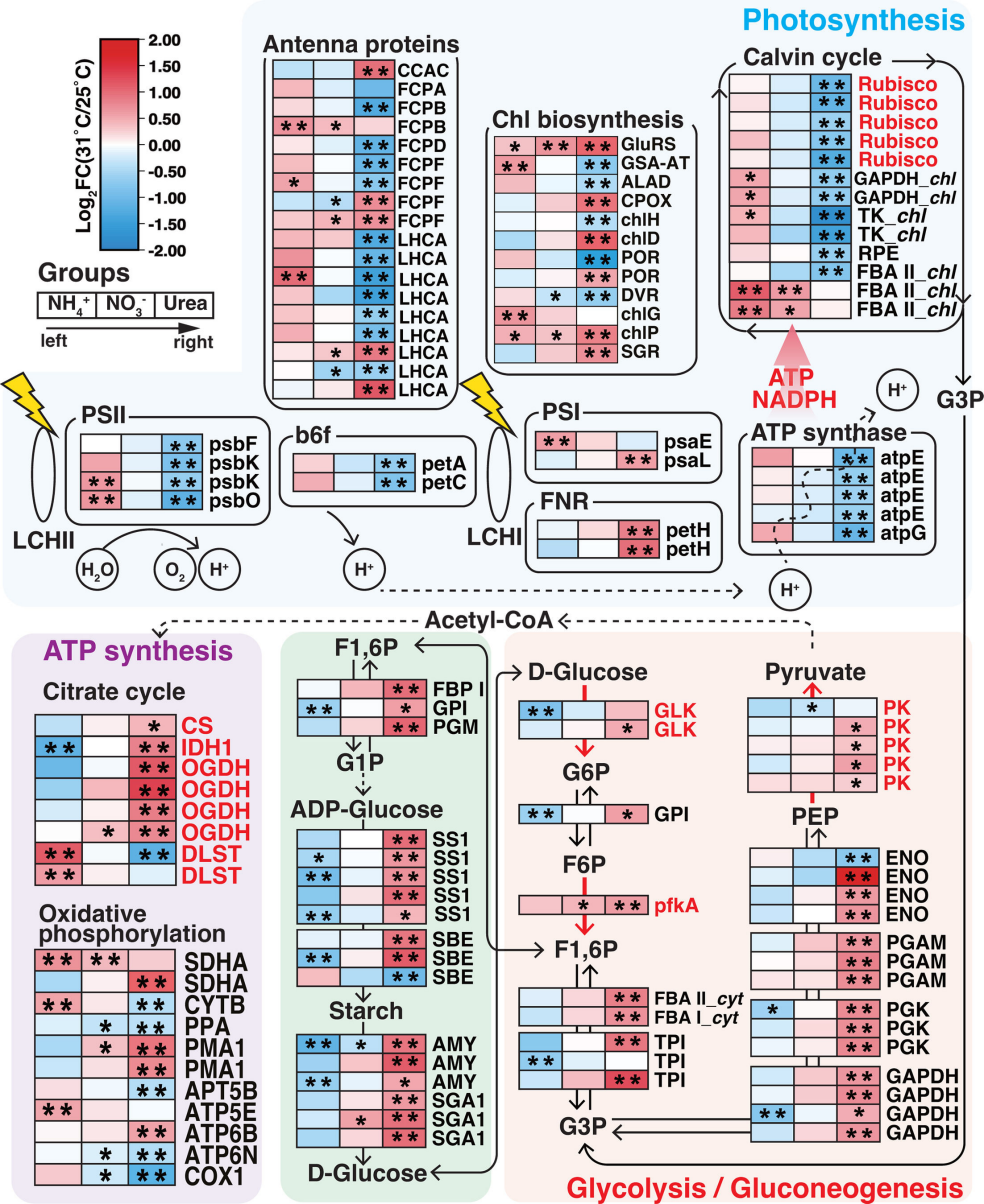
Heatmap of HS-DEGs involved in photosynthesis, carbon fixation, glycolysis/gluconeogenesis, starch metabolism, and lipid metabolism. The fill color of the rectangles from left to right represents the log2 fold change (log2FC) of 31°C compared with 25°C. HS-DEGs are marked as two asterisks inside rectangles, while those with Q-value < 0.05 but with 2/3 ≤ FC(31°C/25°C) ≤3/2 are marked as one asterisk. Metabolite abbreviations: G6P, glucose 6-phosphate; F6P, fructose 6-phosphate; F1,6P, fructose 1,6-biphosphate; G3P, glyceraldehyde 3-phosphate; PEP, phosphoenolpyruvate; G1P, glucose 1-phosphate; TAG: triacylglycerol. Detailed gene descriptions can be found in Table S4.

Energy metabolism pathways were largely upregulated under HS; differences occurred in specific genes upregulated between N types. Succinate dehydrogenase (ubiquinone) flavoprotein subunit (SDHA) involved in ATP synthesis was consistently upregulated under HS in all N groups. HS downregulated glucokinase (GLK), which catalyzes the irreversible reaction in glycolysis in the NH_4_^+^ group. Conversely, most HS-DEGs related to polysaccharide metabolism and glycolysis/gluconeogenesis pathways, including glycolysis-specific enzymes GLK, 6-phosphofructokinase 1 (pfkA) and pyruvate kinase (PK), were upregulated in the Urea group. The NO_3_^-^ group under HS exhibited enhanced transcription of pfkA (FC < 1.5). Under HS, key enzymes that catalyze the irreversible reactions in the citrate cycle were upregulated in the Urea group, while oxidative phosphorylation genes showed consistent upregulation in the NH_4_^+^ group (Fig. 5).

**Fig 6:**
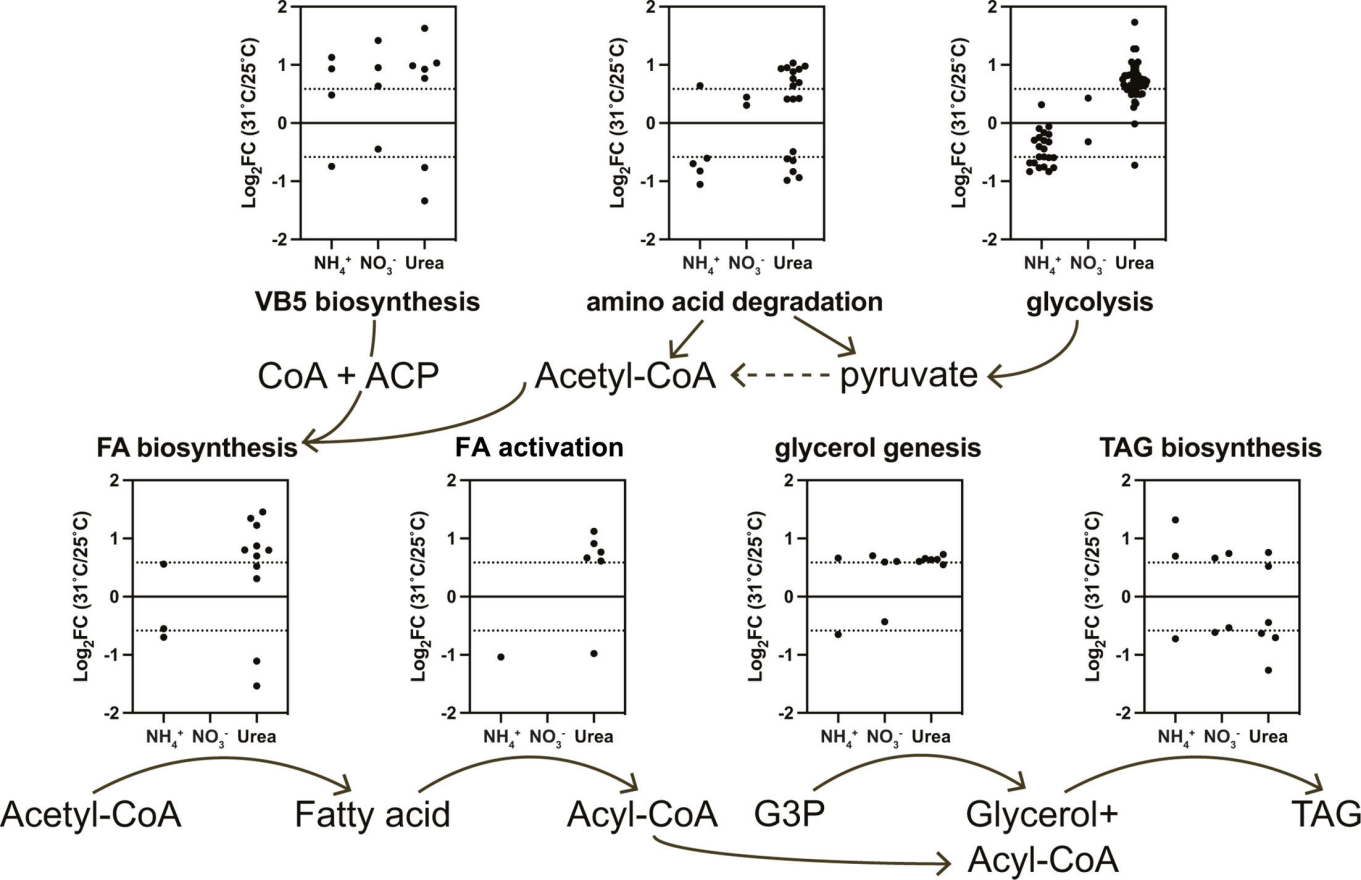
Lipid biosynthesis-related genes significantly regulated under HS. Every dot represents one gene. Dash lines indicate thresholds of HS-DEG where FC(31°C/25°C) =3/2 or 2/3. Metabolite abbreviations: CoA, coenzyme A; ACP, acyl carrier protein; G3P, glyceraldehyde 3-phosphate; TAG, triacylglycerol. Detailed gene descriptions can be found in Table S4.

For lipid metabolism, in the NH_4_^+^ group, HS downregulated genes serving in fatty acid biosynthesis and activation, but upregulated two HS-DEGs involved in triacylglycerol (TAG) biosynthesis. Transcription of glycerol biosynthesis genes was enhanced by HS in the NO_3_^-^ group. In the Urea group, HS upregulated genes associated with fatty acid and glycerolipid biosynthesis, including acetyl-CoA carboxylase (ACCase) and S-malonyltransferase (FabD) for fatty acid biosynthesis, acyl-CoA synthetase (ACSL) for activating fatty acids into long-chain acyl-CoA, and alcohol dehydrogenase (ADH) and aldehyde reductase (AKR1B) for glycerol biosynthesis from G3P and acyl-CoA. Branch-chain amino acid (BCAA) degradation and pantothenate biosynthesis also provide precursors for fatty acid biosynthesis, i.e., acetyl-CoA, pyruvate, acyl carrier protein (ACP), and coenzyme A (CoA). Most HS-DEGs involved in BCAA degradation were upregulated in the Urea group but were either downregulated or unchanged in the NH_4_^+^ and NO_3_^-^ groups. In the process of pantothenate biosynthesis, a gene encoding ketopantoate reductase (KPR) was highly expressed and upregulated under HS in all N groups (FC > 1.7). Other HS-upregulated genes included 3-methyl-2-oxobutanoate hydroxymethyltransferase (panB) in the Urea group, and dihydroxy-acid dehydratase (ilvD) and phosphopantothenate—cysteine ligase (PPCS) in NH_4_^+^ and NO_3_^-^ groups (Fig. 6).

### Transcriptional response of ROS scavenging

**Fig 7:**
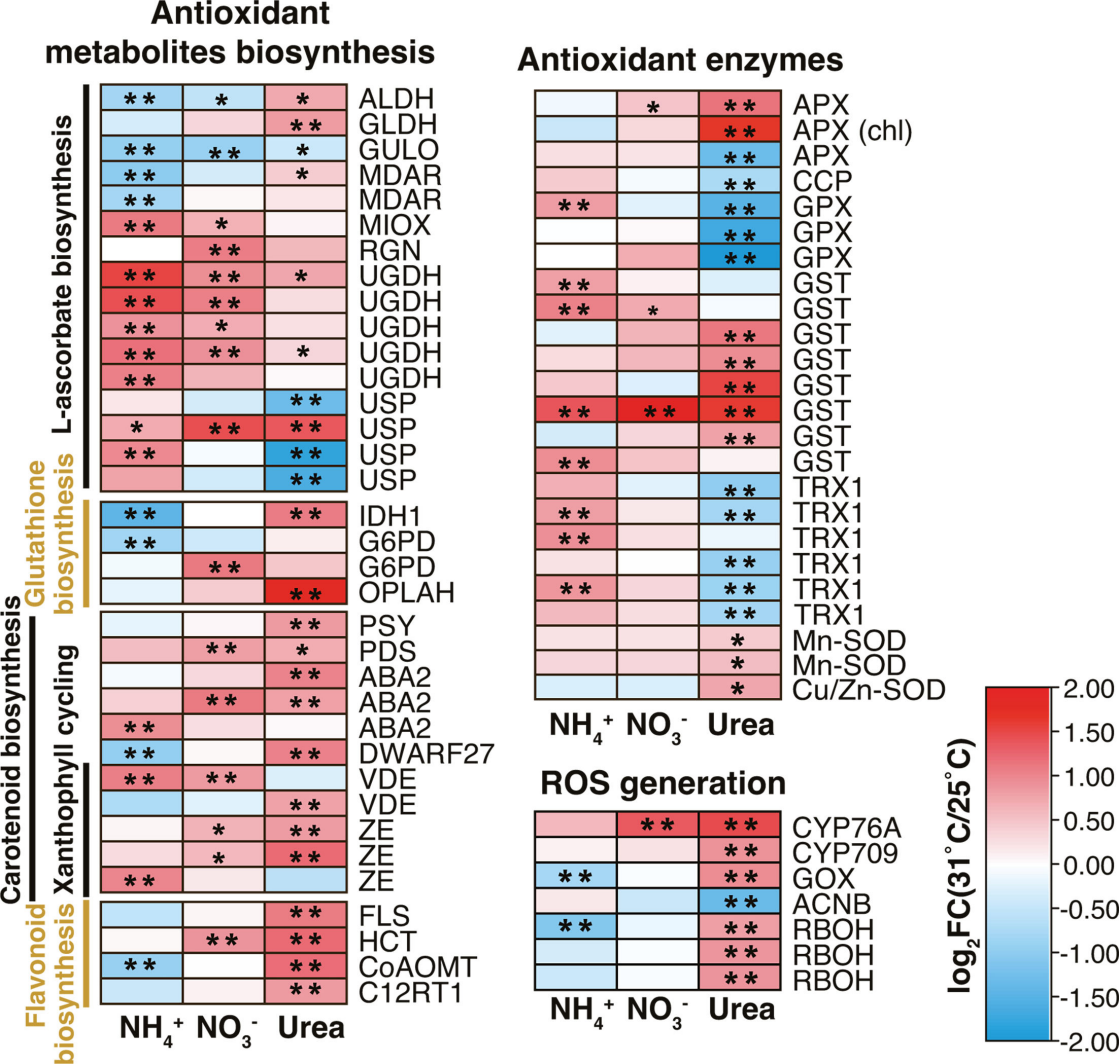
Heatmap of HS-DEGs involved in ROS homeostasis and cross-plasma membrane transport. HS-DEGs are marked as two asterisks inside rectangles while those with Q-value < 0.05 but with 2/3 ≤ FC(31 °C/25 °C) ≤3/2 are marked as one asterisk. Detailed gene descriptions can be found in Table S5.

Most genes involved in ROS scavenging were upregulated under HS in all three nitrogen groups. HS-DEGs involved in L-ascorbate biosynthesis were mostly upregulated in the NH_4_^+^ and NO_3_^-^ groups, while those in flavonoid biosynthesis were mostly upregulated in the NO_3_^-^ and Urea groups. HS caused upregulations of antioxidant enzymes in all N groups, but types of upregulated enzymes varied. Ammonium-grown cells upregulated one guaiacol peroxidase (GPX), four glutathione S-transferase (GST), and three thioredoxin 1 (TRX1) genes under HS. In the Urea group, expressions of ascorbate peroxidase (APX) and GST encoding genes were induced by HS, while GPX and TRX1 genes were downregulated. Only one upregulated HS-DEG encoding GST enzyme was detected in the NO_3_^-^ group (Fig. 7).

### Transcriptional response of photoprotection

Carotenoid and xanthophyll pigment biosynthesis genes were mostly upregulated under HS in all N groups, including violaxanthin de-epoxidase (VDE) and zeaxanthin epoxidase (ZE) crucial in the β-carotene-derived xanthophyll cycle, 15-cis-phytoene synthase (PSY) and 15-cis-phytoene desaturase (PDS) that catalyze lycopene synthesis (precursor of α-carotene and β-carotene) (45, 46).

### Transcriptional response of photosynthate transporters

Under HS, putative plasma membrane transporters showed mixed regulation across N groups. Among these, one gene related to glucose exporting (solute carrier family 50, SLC50/SWEET) was upregulated in the NH_4_^+^ group (FC = 1.73) but downregulated in the Urea group (FC = 0.40). Genes encoding nodulin 26-like intrinsic protein (NIP) with glycerol transport capacity were downregulated in the Urea group but remained unchanged in other N groups (Table S6).

## DISCUSSION

Previous studies have suggested that different forms of nitrogen exert differential impacts on the thermal tolerance of corals (13, 27). A study reported that the uptake of urea by Symbiodiniaceae *Durusdinium glynnii* could alleviate thermal stress response compared with nitrate (47). However, the exact mechanism of the interactive effects of N utilization and thermal stress on Symbiodiniaceae and coral survival remains obscure. The present study examined physiological, biochemical, and transcriptomic responses to different N types on the top of heat stress to shed light on the trend of responses and underlying molecular mechanisms.

### Common responses to HS under all N conditions: cell division and photosynthesis suppression, and lipid storage, energy metabolism and oxidative stress elevation

#### Suppression of cell division and photosynthesis by HS

The population growth of cultured *C. goreaui* was severely suppressed at 31°C (Fig. 1A and B), consistent with previous studies on cultured Symbiodiniaceae (e.g., 48–52). However, some studies have reported an increased mitotic index (MI) of Symbiodiniaceae *in hospite* with coral at high temperatures (31°C–34°C) (e.g., 18, 53–56). This disprecancy might stem from increased ammonium supply from coral amino acid catabolism under HS (18), compensating for HS inhibition and supporting faster *in hospite* algae reproduction. This aligns with findings by Zhou et al. (52) where cultured *C. goreaui* gained higher cell yield under N-replete-high-temperature condition than under N-deplete moderate-temperature conditions. Fujise et al*.* (49) also suggested that MI measurements only capture data at the culture endpoint, which may explain the inconsistency.

Symbiodiniaceae cell cycles were found to be arrested in the G1-phase under P-limitation and HS (49, 57, 58). However, in this study, we observed an increased S-phase cell proportion and decreased G1-phase cell proportion of *C. goreaui* after 9 days of heat treatment, accompanied by slow but not completely halted cell proliferation, along with enlarged cell size and increased cellular contents of carbon, nitrogen, sugar, starch, lipid, amino acid, protein, and RNA, resembling features of S-phase cells (Fig. 1A, B, C, D, G through N; Fig. S2) (59). This is similar to studies on phytoplankton *Emiliania huxleyi*, which experienced prolonged S-phase with increased cell size and cellular granularity under *P* limitation and bacterial quorum-sensing signal exposure (60, 61). The cell cycle arrest was apparently underpinned by the significant downregulation of cyclin A genes (CYCA) across all N groups (although with 2/3 < FC < 1), as CYCA downregulation can lead to decreases in cyclin-dependent kinase activities, resulting in cell cycle arrest (62, 63).

Photosystem II (PSII) is the most thermosensitive site of photosynthetic apparatus (64, 65). Changes in the PSII maximum photochemical yield (*Fv/Fm*), real quantum yield (Y(II)), non-photochemical quenching yield (Y(NPQ)) and relative electron transport rate (rETR) serve as indicators of thermal stress (66, 67). Decreased *Fv/Fm* under HS as documented in *C. goreaui* (Fig. 2A) has been widely reported in Symbiodiniaceae (68–74). Increased Y(NPQ) and Y(NO), but decreased Y(II) after heat exposure (Fig. 2F through H), suggests that excess excitation energy induced and overwhelmed the photoprotection capacity, leading to closure of PSII reactive centers and reduced photochemical quantum yield (75, 76).

Photoprotection was potentially activated under HS, as evidenced by the concordant upregulation of xanthophyll cycle genes VDE and ZE (Fig. 7). Xanthophyll pigments are correlated with transthylakoidal ΔpH quenching (qE), the main component of NPQ (77–79). The lower rETRmax and repressed rETR curves in heat-treated groups also indicated over-reduction of chloroplast electron transport chain (Fig. 2E; Fig. S3), partly due to decreased carbon assimilation in the Calvin cycle (80, 81) resulting from the typically decreased CO_2_ affinity of Rubisco under HS (65, 82, 83).

HS causes decreases in carbonic anhydrase (CA) activity in Symbiodiniaceae (84), affecting inorganic carbon-concentrating mechanism (CCM) crucial for the low CO_2_ fixing efficiency of form II Rubisco in dinoflagellates (85, 86). Upregulated *C. goreaui* CA expressions under HS in all N groups (Fig. 4) suggest the potential of enhancing CCM under HS to promote photosynthetic carbon fixation.

No change in D1 protein abundance under HS was observed in *C. goreaui* (Fig. 2N and O), which aligns with previous studies on other Symbiodiniaceae strains (74, 87, 88). These suggest that a unique mechanism potentially exists in Symbiodiniaceae where the regulation of other photosystem proteins, but not D1 protein, plays more important roles in coping with HS (74).

#### Lipid storage, energy metabolism and ROS management

*C. goreaui* under HS exhibited a significant three- to sixfold neutral lipid (triacylglycerol, TAG) increase (Fig. 1J and N), accompanied by upregulated glycerol biogenesis genes (Fig. 7). Lipid accumulation is a widely observed symptom for symbiotic zooxanthellae under HS (89–93). These stored lipids can provide carbon and energy for microalgae regrowth and reproduction when the environment is suitable (94). Upregulation of Vitamin B5 biosynthesis-related genes under HS in all N groups, including the highly expressed KPR, provided additional precursor for fatty acid synthesis (Fig. 6) (95–97). This indicates that increased lipid biosynthesis, facilitated by vitamin B5 accumulation, is important for *C. goreaui* under HS in all N conditions tested.

Similar to other phytoplankton species, including dinoflagellates (98–101), *C. goreaui* enhanced energy metabolism under HS, as indicated by upregulated glycolysis, TCA cycle, and mitochondrial oxidative phosphorylation (Fig. 5). Succinate dehydrogenase (ubiquinone) flavoprotein subunit (SDHA), which catalyzes the oxidation of succinate and produces electrons for the mitochondrial electron transport chain (102), was consistently upregulated under HS across N groups (Fig. 5), alluding to its vital role in heat-responsive energy metabolism in *C. goreaui*. These adjustments provide *C. goreaui* with sufficient energy to cope with HS. These factors, along with inhibited photosynthesis of *C. goreaui* under HS, could lead to reduced photosynthate available for translocation to coral during symbiosis. This parallels a previous study suggesting decreased carbon translocation from symbionts to corals under HS due to reduced photosynthesis and increased carbon storage, as reflected by proteomic data (93).

As an inevitable byproduct of aerobic metabolism, ROS can cause significant oxidative damage to cells when their production exceeds the counterbalancing capacity of antioxidants (103, 104). Exposure to elevated temperatures can cause excess ROS build-up in Symbiodiniaceae, potentially leading to coral tissue damage and alga expulsion due to H_2_O_2_ permeation across the symbiosome membrane (105–107). Studies had revealed enhanced ROS production and antioxidant enzyme activities under HS in different cultured Symbiodiniaceae species (72, 108, 109). Here, we found that *C. goreaui* consistently enhanced total antioxidant capacity (ferric-reducing power) for more than twofold under HS across all N groups (Fig. 2L). This, combined with upregulated genes involved in antioxidative metabolite (e.g., carotenoid, ascorbate, flavonoid) biosynthesis, indicated upregulation of non-enzymatic antioxidative capacity for ROS scavenging. Types of HS-upregulated antioxidant enzymes varied across N groups, indicating complex N-dependent regulations (Fig. 7).

#### Nitrogen utilization

In diatom-dominated phytoplankton populations, uptake rates of nitrate decreased with increasing temperature (7°C–25°C), but ammonium and urea uptake rates generally increased (110). We observed similar trends during early stage of culture (day 0 to day 3) where ammonium and urea but not nitrate were depleted faster under HS. Different trends thereafter could be attributed to lower ammonium or urea concentration in HS groups, leading to generally insignificant effect of HS on N depletion (Fig. 1E and F). *C. goreaui* generally showed upregulated ammonium and nitrate transporters (AMT, NRT, NRT2.5), and downregulated urea transporter (DUR3) under HS in all N groups (Fig. 4), which were also observed in *in hospite* Symbiodiniaceae (dominated by ITS2 type C) after 192 h of heat treatment (111), indicating a universal transcriptional response to HS regardless of nitrogen utilization or living states.

### Urea exacerbating detrimental effects of HS

Urea utilization can potentially reduce photosynthate export under HS. Firstly, urea-treated cells exhibited marked reductions in photosynthesis gene expression and Rubisco transcript and protein abundances (Fig. 2N, O and 5), suggesting suppression of carbon fixation. Transcriptionally prompted photorespiration (Table S4) indicated enhanced oxygenation of Rubisco and concurrent H_2_O_2_ production (65, 112, 113). Secondly, lipid storage under HS appeared to be reinforced when urea was the sole nitrogen source. Under HS, urea-grown cells showed double the neutral lipid content compared with the NH_4_^+^ and NO_3_^-^ groups, accompanied by upregulated genes for fatty acid biosynthesis, activation, glycerol biosynthesis, and BCAA degradation, which replenishes the acetyl-CoA pool for phytoplankton lipid biosynthesis (114, 115) (Fig. 6). Potential reduced exportation of glucose and glycerol, key photosynthates provided by Symbiodiniaceae to corals (34, 116, 117), is indicated by downregulated SWEET and NIP genes responsible for their transport in Symbiodiniaceae (118, 119) and symbiotic soybean cells (120), respectively (Table S6). Together, these findings suggest that urea enrichment can exacerbate photosynthate supply detention from *C. goreaui* to symbiotic corals under HS.

Urea assimilation also led to higher ROS stress under HS. Only the Urea group cells exhibited a significant increase in cellular ROS content under HS (Fig. 2I). The increased ABTS·^+^ scavenging capacity and total ferric-reducing power (Fig. 2J and L) indicated that the excess ROS resulted from more severe photorespiration and protein refolding, and not reduced antioxidant capacity. Additionally, urea-grown cells upregulated respiratory burst oxidase family (RBOH) under HS (Fig. 7), which produces superoxide anion (O_2_^−^) at plasma membrane (121, 122), converting to H_2_O_2_ and potentially reaching coral cells during symbiosis (121–123). Thus, urea enrichment could exert more detrimental effects on symbiosis during HS due to higher intra- and extracellular ROS generation.

In the Urea group, downregulated GS, GSIII, and GOGAT-NADPH under HS, alongside upregulated carbamoyl-phosphate synthase II (CPSII) (Table S3), altered nitrogen metabolism. Suppressed GOGAT may lead to α-ketoglutarate (AKG) accumulation, scavenging H_2_O_2_ and enhancing antioxidant (124, 125). CPSII-derived carbamoyl phosphate could be directed to *de novo* pyrimidine biosynthesis (122), which suggested DNA damage. These were evidented by enriched upregulated DNA replication pathway, upregulated pyrimidine and purine metabolism genes and DNA damage response genes (Fig. 3D; Table S7). These indicates that under HS, urea-assimilating *C. goreaui* prioritizes nitrogen allocation towards pyrimidine metabolism for DNA repair, saving AKG for the TCA cycle and ROS scavenging (Fig. S5).

Under HS, the Urea group showed numerous downregulated ribosomal subunits and spliceosome snRNPs, indicating suppressed pre-mRNA maturation and nascent polypeptide biosynthesis. Upregulated HSPs suggested accumulated misfolded proteins and aided in protein refolding (126–131). This pressure increased energy metabolism and ROS levels due to ATPase activity of chaperones and ROS generation during disulfide bond formation (132), as evidenced by upregulated protein disulfide-isomerase (Table S3). Reduced total protein translation but enhanced secretory protein processing in ER under HS likely led to reduced intracellular protein biosynthesis, including photosynthetic machinery, as evidenced by decreased Rubisco content and photosynthetic gene transcriptions in the Urea group (Fig. 4 and 5).

Finally, urea utilization in combination with heat stress caused cell cycle arrest more severely than *C. goreaui* cells cultured with nitrate or ammonium. The Urea group displayed transcriptional increases related to nucleotide metabolism and DNA replication or damage response as well as inhibitory cell cycle regulators, such as as WEE1-like protein and cell division cycle 20 (CDC20). WEE1 serves as cell division inhibitors through inhibition of CDKA activity (133), and CDC20 promotes mitosis exit through cyclin degradation (134).

### Two sided effects of ammonium under HS

Ammonium utilization alleviated cell reproduction inhibition and cell cycle arrest under HS, as indicated by a significantly higher population growth rate and a smaller increase in the proportion of S-phase cell (Fig. 1A and D). Higher Symbiodiniaceae density in coral tissue in ammonium-added culture than in nitrate-added culture under both 25°C and 30°C was also recently reported (135). Since coral bleaching involves decreased symbiotic algae density in coral tissue, the assimilation of ammonium should benefit symbiosis by supporting algal population growth.

**Fig 8:**
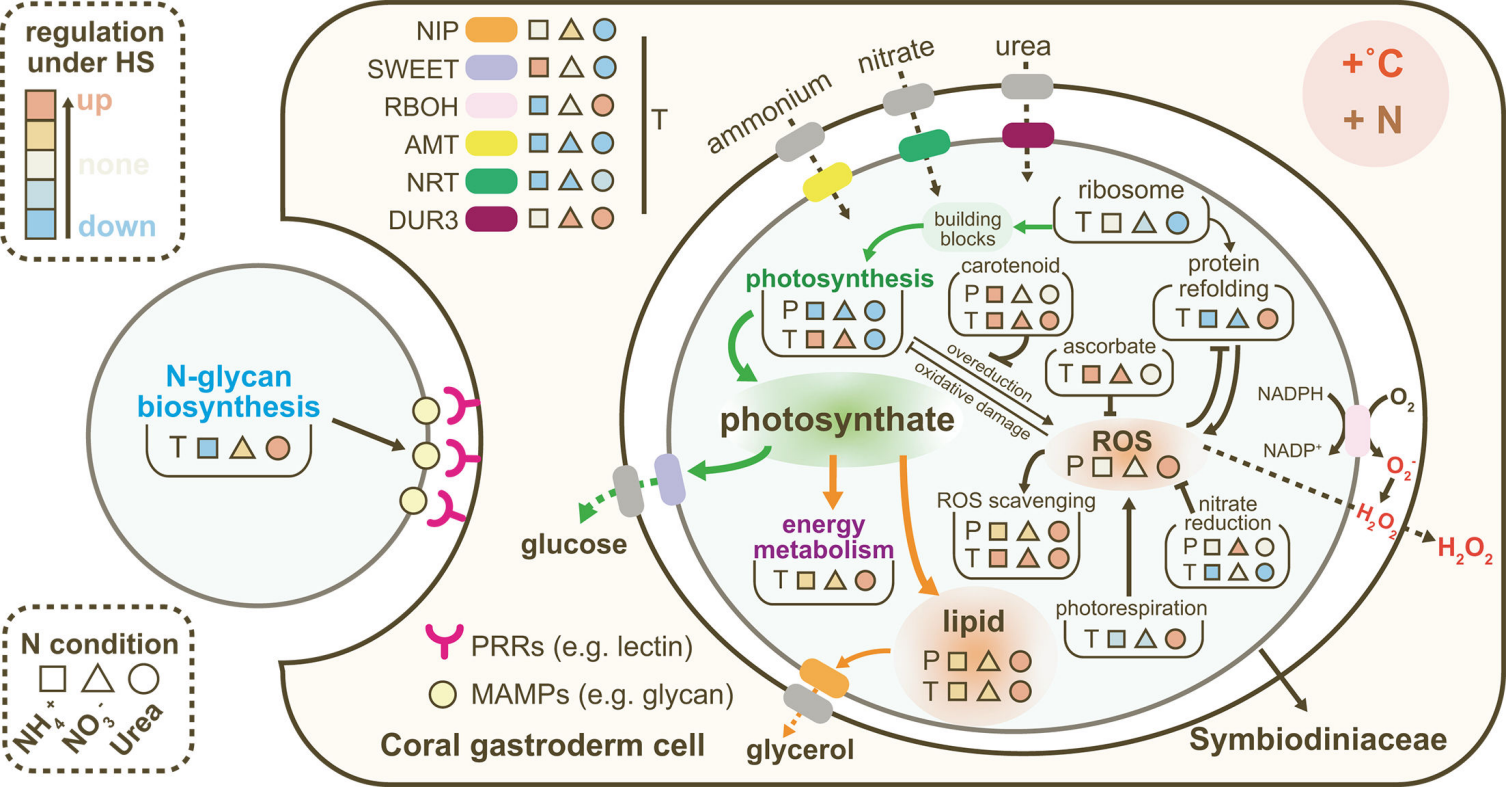
Schematic of potential impacts of heat stress in *C. goreaui* on symbiosis with coral under different types of N. Arrows represent directions of material flux and T-bar arrows indicate inhibited or alleviated effects. Green arrows indicate decreased material flux, while orange arrows indicate increased material flux. The letter “T” and “P” denote conclusions supported by transcriptome data and physiological data, respectively.

Ammonium supply could benefit photosynthesis by increasing pigment biosynthesis under HS. Genes conferred chlorophyll biosynthesis (e.g., glutamate-1-semialdehyde 2,1-aminomutase, chlorophyll/bacteriochlorophyll *a* synthase) (136, 137) were upregulated under HS in the NH_4_^+^ group, correlating with increased chlorophyll *a* and *c* contents (albeit without statistic significance) (Fig. 2 and 5). In addition, the increase in carotenoid content under HS was significant only in the NH_4_^+^ group, despite upregulation of carotenoid biosynthesis-related HS-DEGs in all N groups (Fig. 2D and 8). Carotenoids can deactivate triplet chlorophyll (^3^Chl⁎) and singlet oxygen (^1^O_2_⁎) and play an important role in photoprotection (138). Thus, ammonium-grown cells might have higher photoprotection potential and consequently less chloroplast ROS production under HS due to the increased carotenoid content.

However, ammonium enrichment might reduce coral–Symbiodiniaceae recognition under HS. Interactions between algal glycans and host pattern recognition receptors (PRRs) are crucial for recognition and infection during cnidarian–Symbiodiniaceae symbiosis (4, 139–141). N-acetyl and mannose residues, which bind PRRs, are prominent of Symbiodiniaceae cell-surface glycans (141). Similar to *Fugacium kawagutii* (139), enzymes involved in the final steps of glycan biosynthesis (i.e., MAN2, MGAT2, MGAT4, MGAT5, MGAT4C) were absent in our transcriptome data of *C. goreaui*, likely resulting in terminally mannose-abundant glycans ((GlnNAc)4(Man)5(Asn)1) for lectin binding (139, 142). Transcriptionally increased N-glycan production in urea- and nitrate-grown cells might aid in symbiosis re-establishment with corals after the bleaching, but for ammonium-assimilating cells, the recognition might be more challenging due to less N-glycan synthesis (Fig. 4).

### Potential role of nitrate reduction in facilitating resilience against HS

Nitrate-grown cells exhibited the fewest HS-DEGs among the three N types examined (Fig. 3B), indicating possible acclimation-promoting effects of nitrate. This beneficial effect may be related to nitrate reduction, which requires reducing power (NADH or NADPH) and electrons. HS-induced nitrate reductase (NR) activity was observed in nitrate-grown cells, but remained unchanged with downregulated transcription of NR and NiR when nitrate was absent (in the NH_4_^+^ or Urea group) (Fig. 2M and 4). Nitrate reduction could serve as an important sink for excess reductant, protecting against chloroplastic over-reduction and ROS damage (143–146). Increased nitrate reduction, along with higher rETR and lower ROS level under 31°C in the NO_3_^-^ group (as shown in Fig. S3; Fig. 2I), may contribute to HS resilience.

Lower ROS level in the NO_3_^-^ group may also relate to higher ascorbate biogenesis under HS, which is an important antioxidant (147). Both NH_4_^+^ and NO_3_^-^ groups showed enriched upregulated pentose and glucuronate interconversion (ko00040) and ascorbate and aldarate metabolism pathways (ko00053) (Fig. 3D). UDP-sugar pyrophosphorylase (USPase) and UDP-glucose 6-dehydrogenase (UGDH), present in both pathways, were among the most upregulated HS-DEGs (Table S4). Both enzymes catalyze the biosynthesis of UDP-sugars, essential precursors for plant cell wall polysaccharides, as well as glycoproteins and glycolipids (148, 149), and are involved in ascorbate biosynthesis through the uronic pathway in animals, a pathway also predicted in plants (150, 151).

### Conclusion

Our study integrated physiological, biochemical, and transcriptomic data to shed light on how heat stress interacts with the chemical form of nitrogen nutrient to impact the physiology of coral symbiont algae and underlying molecular mechanisms. Our results reveal common responses of *C. goreaui* to HS (e.g., uniformly downregulated cell reproduction and photosynthesis, upregulated antioxidant capacity, and lipid content) but with different extents across N groups. More importantly, our data indicate that nitrate-grown cells showed the best HS acclimation and resilience, which might be partially due to activated nitrate reduction and ascorbate biogenesis. Enhanced carotenoid and ascorbate quenching to mitigate ROS accumulation might enable ammonium-assimilating cells to endure HS. Cells under ammonium supply showed higher population growth, but they might lower the recruitment probability due to decreased microalgae N-glycan biosynthesis. In comparison, urea eutrophication appeared to be the most detrimental to symbiosis under HS, as urea-grown cells under HS showed greater reduction of carbon fixation and photosynthate export but more severe ROS production, which if happening *in hospite* would cause damage to the host (Fig. 8). These findings underscore the need to consider interactive effects of N type and elevated temperature in coral conservation, with particular attention required for urea pollution.

## MATERIALS AND METHODS

### Algal culture and experiment setup

*Cladocopium goreaui* (strain CCMP2466) was obtained from the National Center of Marine Algae and Microbiota (NCMA) and cultured in L1 medium (152) at 25°C, illuminated with a photon flux of 100 µmol photons m^−2^ s^−1^ under a 14:10 h light: dark cycle. Cultures were maintained in N-deprived L1 mediums for depletion of nitrate for 20 days, with antibiotic cocktails of 50 µg mL ^−1^ streptomycin, 50 µg mL ^−1^ kanamycin, and 100 µg mL ^−1^ ampicillin added to inhibit the influence of bacteria on the experiments.

As high concentrations of ammonium were reported to cause toxic effects on dinoflagellates, including Symbiodiniaceae (36, 153), an ammonium toxicity testing experiment was carried out before formal experiments. In the present study, N-starved algal cultures were inoculated into fresh media containing NH_4_Cl at concentrations of 882, 441, 220, and 110 µM separately, and into control media containing 882 µM NaNO_3_. Results indicated that population growth and cellular chlorophyll *a* content of *C. goreaui* were markedly suppressed both under 882 and 110 µM of ammonium compared to 882 µM nitrate (MANOVA and one-way ANOVA, *P* < 0.05), likely due to high ammonium toxicity and the quick exhaustion of medium ammonium. Cells grown under 441 and 220 µM ammonium achieved comparable cell yields and chlorophyll *a* contents to the control group (MANOVA and one-way ANOVA, *P* > 0.05). To avoid early depletion of medium ammonium, further experiments used an initial nitrogen nutrient concentrations of 441 µM (Fig. S6).

In the formal experiment, the N-starved algal cultures were transferred into six different groups, including cultures supplied with three different states of nitrogen (ammonium, nitrate, and urea at the initial concentrations of 441 µM) under two different temperature conditions (25°C and 31°C). Each group comprised three replicates.

### Measurement of cell growth, cell size and cell cycle profile

Samples of 2 mL algal suspension were collected daily throughout the experiment and fixed with 2% Lugol’s solution. One milliliter of the sample was used for cell diameter measurement using the software ZEN 2012 (blue edition) with the microscope (ZEISS Axio Imager 2, Jena, Germany). The remaining 1 mL sample was diluted with seawater and used for cell counting with a Sedgwick–Rafter counting chamber (PhycoTech, USA) under the microscope. The specific growth rate was calculated following Shi et al. (154), from linear regression of the natural logarithm of cell concentration data from the exponential growth phase and the corresponding time interval.

For cell cycle analysis, about 10^6^ *C. goreaui* cells from each culture were harvested by centrifugation (5,000×*g*, 10 min, 4°C) about 10 h into light period on day 12 of culture. The pellet was re-suspended and fixed in 1 mL 75% ethanol, and stored at −20°C. The fixed samples were processed as Li et al. (57) to further remove algal pigments and stained with PI, and then tested for DNA-bonded PI fluorescence using a Cell Lab Quanta SC flow cytometer (Beckman Coulter, USA). Cell cycle profiles collected from 20,000 cells in each sample were analyzed using ModfitLT-1 software.

### Measurement of cellular contents of sugar, lipid, amino acid, and protein

Biochemical contents of *C. goreaui* were measured with cells collected on day 12. Total sugar contents were measured using the DNS (3,5-dinitrosalicylic acid) method, following Wu et al. (155) with minor adjustments. About 10^7^ *C. goreaui* cells from each sample were harvested by centrifugation (5,000 × *g*, 10 min, 4°C). The pellets were suspended in 0.6 mL ultrapure water, added with glass beads (1:1 mixed 0.5 and 0.1 mm beads), and homogenized thoroughly using the Fastprep−24 Sample Preparation System (MP Biomedicals, USA). After adding 0.4 mL HCl (6 mol/L) to each sample, the mixtures were incubated at 100°C for 30 min. After neutralizing pH to 7.4 using NaOH solution, 50 µL supernatant extract from each sample was mixted with 100 µL DNS reagent (Yuanye, China) and incubated at 100°C for 5 min. Absorbances at 540 nm (A_sample_) were measured by SpectraMax Paradigm Microplate Reader (Molecular Devices, USA). Another 50 µL extract from each sample was mixed with 100 µL water as a sample control (A_sampleblank_) in order to subtract the absorbance of pigments. Adjusted absorbances (A_adj_ = A_sample_-A_sampleblank_) were used for the total sugar concentration calculation based on a glucose standard curve.

Amino acid contents were measured using the color reaction of α-amino of amino acid with ninhydrin. About 10^7^ *C. goreaui* cells from each culture were harvested by centrifugation (5,000 × *g*, 10 min, 4°C). The pellets were re-suspended in 0.8 mL of regent_1 solution in the Amino Acid Content Assay Kit BC1575 (Solarbio, China), then homogenized as previously decribed. The supernatant extracts after centrifuging were then used for detection following the kit’s protocol. Absorbances at 570 nm were read by SpectraMax Paradigm Microplate Reader. For absorbance adjustment, 10 µL extract from each sample was added to 210 µL water and measured at 570 nm. A 10 µmol/mL cysteine solution was used as a standard.

Protein contents were measured using the bicinchoninic acid (BCA) assay. About 2 × 10^7^ cells from each culture were harvested by centrifugation (5,000 × *g*, 10 min, 4°C) and resuspended in 0.3 mL PBS solution. Cells were homogenized as described above. After centrifugation, the supernatant was transferred to a fresh tube and diluted four times before protein concentration was quantified using the BCA assay kit P0012S (Beyotime, China). Absorbances at 562 nm were read by SpectraMax Paradigm Microplate Reader and used for protein content calculation based on the standard protein curve. To calibrate against background absorbance, solutions containing 20 µL extract from each sample and 200 µL PBS were subjected to the same measurement.

Lipid contents of *C. goreaui* were measured following Li et al. (156). Cells were harvested as described above and stained with the BODIPY 505/515 stain. Fluorescence signal of 20,000 cells was measured for each sample using the Cell Lab Quanta SC flow cytometer (Beckman Coulter, USA) with 488 nm excitation and 525/40 BP emission.

### Measurement of cellular C and N contents and medium nitrogen concentrations

Cellular carbon and nitrogen contents were measured on day 12 following the protocol by Ehrhardt and Koeve (157), with minor modifications. Fifteen milliliters from each culture was filtered through a pre-combusted 25 mm GF/F filter and stored at −20°C. The filters were defrosted and dried in a 55°C oven for over 12 h, fumigated with 1 mol/L HCl, and dried again. The dried filters were then encapsulated with tin foil sheets and combusted in a Vario EL cube elemental analyzer (Elementar, Germany).

To determine nitrogen concentrations in the medium, 10 mL from each sample was filtered through 0.22 µm filters, and the filtrates were stored at −20°C for subsequent analysis. Ammonium, nitrate, and urea concentrations were measured using a spectrophotometer (V-5600, METASH, China), with the indophenol blue method (158), the Griess reaction method with vanadium chloride as reductant (159), and the diacetyl monoxime method (160), respectively.

### Measurement of chlorophyll fluorescence parameters and pigment contents

Two milliliters of algal suspension was collected every 3 days for each culture for photochemistry efficiency (*F_v_/F_m_*) measurement using a Fluorescence Induction and Relaxation Fluorometer System (Satlantic, Canada) after 20 min dark acclimation. Four milliliters of culture from each sample was collected on day 9 and measured for slow kinetics of chlorophyll fluorescence and rapid light curve using the Multi-color PAM fluorometer (WALZ, Germany) after at least 20 min of dark acclimation. Data were recorded and calculated by Pamwin-3 software, and rapid light curve data were further analyzed for non-linear regression based on the previously reported equation (161) using Prism-9 software.

Cellular pigment contents were measured following Li et al. (162). On day 12, about 5 × 10^6^ cells were harvested by filtering culture samples onto 25 mm GF/F membranes. Then, 4 mL of pure methanol was added to immerse the membrane and stored in the darkness at 4°C overnight. After centrifugation, the absorption spectra of the methanol extracts were scanned using a UV-VIS Spectrophotometer (Agilent Technologies, USA). The contents of chlorophyll *a*, chlorophyll *c_2_* and carotenoids were calculated based on A_480_, A_510_, A_632_, A_665_, and A_750_, using the Ritchie equations (163, 164):


$$\begin{array}{rl} & \text{ Chl }a(\mu g/mL)=13.6849\times \left(A_{665}-A_{750}\right)-3.4551\times \left(A_{632}-A_{750}\right)\\ & {Chl\;c}_{2}(\mu g/mL)=-7.0140\times \left(A_{665}-A_{750}\right)+32.9371\times \left(A_{632}-A_{750}\right)\\ & \text{ Carotenoid }(\mu g/mL)=7.6\times \left[\left(A_{480}-A_{750}\right)-1.49\times \left(A_{510}-A_{750}\right)\right] \end{array}$$

### Measurement of cellular ROS level, anti-oxidative capacity, and nitrate reductase activity

These data were measured on day 12 of culture. Intracellular ROS was determined using Reactive Oxygen Species Assay Kit CA1410 (Solarbio, Beijing, China), following the modified manufacturer’s instruction. In brief, about 6 × 10^6^ to 8 × 10^6^ cells from each culture were harvested by centrifugation and re-suspended with 1 mL sterile seawater, then injected with 1 µL DCFH-DA ROS probe and incubated at experimental temperature (25°C or 31°C) for 1.5 h. After that, the cells were washed with sterile seawater thrice and re-suspended in 300 µL sterile seawater. The suspension was transferred into a 96-well plate (three technical replicates, each 100 µL/well), and measured for fluorescence signal by SpectraMax Paradigm Microplate Reader at 488 nm excitation and 525 nm emission.

DPPH (2,2-diphenyl-1-picrylhydrazyl) radical-scavenging activity was tested following previous reports (165, 166) with minor adjustments. After the harvesting of about 10^7^ cells from each culture by centrifugation, the cells were resuspended in 1 mL pure methanol and homogenized as described previously. Then, 20 μL of extract supernatant was added to 180 µL methanolic DPPH solution (0.02 mg/mL). The samples were mixed thoroughly in a 96-well plate and read for absorbance at 517 nm after 30 min using the SpectraMax Paradigm Microplate Reader. The DPPH radical scavenging ability was calculated as the following equation:


$$Scavenging\;effect\;(\%)=[1-(A_{sample}-A_{MeOHblank}-A_{sampleblank})/A_{control}]\times 100$$

A_sample_: absorbances of methanolic DPPH solution with sample extracts; A_MeOHblank_: the absorbance of pure methanol; A_sampleblank_: absorbances of extracts in methanol (20 µL extract from each sample and 180 µL methanol, for subtraction of the absorbance of pigments); A_control_: the absorbance of the methanolic DPPH solution.

Trolox equivalent antioxidant capacity (TEAC) assay was applied to assess the ABTS^·+^ radical scavenging capacity (167, 168). About 10^7^ harvested cells (by centrifugation) from each culture were resuspended and lysed into 0.4 mL PBS solution with glass beads using the Fastprep System. Then, 10 μL extract supernatant from each sample was tested following the T-AOC assay kit S0121 (Beyotime, China) protocol. Radical scavenging capacity was expressed as Trolox equivalent antioxidant capacity (TEAC) calculated from the Trolox standard curve. Absorbances of extracts in PBS were measured to account for pigment interference.

Ferric-reducing antioxidant power (FRAP) assay was carried out to measure the total reducing capacity of electron-donating antioxidants of *C. goreaui* cells (168, 169) using another T-AOC assay kit BC1315 (Solarbio, China). Cell extracts were prepared by breaking about 10^7^ cells from each culture in 0.4 mL extraction solution with Fastprep System, followed by centrifugation. Then, 12 μL supernatant extract from each sample was tested following kit protocol, with reaction duration extended to 20 min.

Nitrate reductase activity was measured using the nitrate reductase activity kit (BC0085, Solarbio, China). About 10^7^ cells from each culture were lysed in 0.4 mL extraction solution by beads-beating in the Fastprep System, followed by centrifugation. Then, 80 μL supernatant extract from each sample was used for testing following kit protocol.

### Western blot analysis of Rubisco and PSII D1 protein

Western blots were performed using free-stain imaging technology (Bio-Rad, USA) with cells collected on day 12. Then, 8 μg total protein from each protein extract was loaded onto a 12% stain-free polyacrylamide gel (TGX Stain-Free FastCast Acrylamide Kit, 12% #1610185, Bio-Rad, USA). After the electrophoresis performed by Mini-PROTEAN Tetra Cell (Bio-Rad), the gel was activated and imaged immediately on a ChemiDoc XRS + System (Bio-Rad). Proteins were then transferred to a polyvinylidene fluoride (PVDF) membrane using the Trans-Blot Turbo Transfer System (Bio-Rad, 25V, 1A, 30 min). The membrane was blocked for 1 h in TBST-milk buffer (10% skim milk powder in 1× Tris-buffer saline solution with 0.1% v/v Tween-20), then probed with dinoflagellate Form II Rubisco antiserum (170) (1:10,000 diluted) and a secondary antibody (HRP-labeled goat anti-rabbit IgG, A0208, Beyotime, 1:1,000 diluted), visualized with ECL reagents (Clarity Western ECL Substrate, Bio-Rad) using the ChemiDoc XRS + System. The membrane was then washed with Western Stripping Buffer (P0025B, Beyotime) for 10 min, re-probed with anti-D1 protein (Rabbit anti-PsbA antibody, Agrisera, Sweden, 1:20,000 diluted) and analyzed. Bands of Rubisco and PsbA were normalized to total protein lanes and analyzed with ImageLab 6.1.

### RNA isolation

Approximately 0.5 × 10^7^–1×10^7^ cells from each culture were harvested on day 12 of the culture by centrifugation (5000 ×*g*, 10 min, 4°C), and cell pellets were resuspended in 1 mL TRIzol Reagent (Invitrogen, USA) and stored at −80°C. Total RNA extraction followed a previously reported method (171) with slight modification. Briefly, after defrosting on ice, cells were thoroughly lysed using a Fastprep System with glass beads (1:1 mixed 0.5 mm and 0.1 mm beads, 6 M/s, 50 s, three cycles). The released RNA was extracted using chloroform and further purified using Direct-zol RNA Miniprep kit R2052 (Zymo Research, Orange, USA). The quality and concentration of RNA samples were determined using a NanoDrop ND-2000 Spectrophotometer (Thermo Scientific, USA) and Equalbit RNA BR Assay Kit (Vazyme, China).

### RNA-seq and gene expression analysis

A total of 18 RNA-seq libraries were constructed using DNBSEQ platform (BGI Genomics Co., Shenzhen, China). Raw sequencing data were filtered with SOAPnuke v1.4.0 (172) to remove reads containing adapters, unknown base (>5%), and low-quality bases. Clean data in FASTQ format were mapped to the published *C. goreaui* genome and assembled unique genes (140) using HISAT v2.1.0 (173) and Bowtie2 v2.2.5 (174), respectively. New transcripts were reconstructed using StringTie v1.0.4, followed by Cuffmerge to integrate the assembled transcripts and to select the new transcripts by comparing the integrated transcripts to the reference annotation. New transcripts predicted to have the protein-coding potential (performed by CPC v-.0-r2) were added to the reference gene sequences, based on which the subsequent analysis was conducted.

Principal component analysis (PCA) was performed on the BIC website (175). Gene expression level was calculated using RSEM v1.2.8 (176). Differential gene expression analysis was conducted by DEseq2 (177), and genes with fold change > 1.5 and Q-value < 0.05 between 25°C and 31°C treated groups under the same nitrogen condition were considered heat-stress induced DEGs (HS-DEGs). Heat maps and Venn diagrams of HS-DEGs were drawn using TBtools (v1.0) (178). HS-DEGs shared among all three N groups with the same regulation trends under heat stress were defined as coHS-DEGs. GO functional clustering graph of concordant HS-DEGs was analyzed using the R package simplifyEnrichment (v1.8.0) based on semantic similarities of GO terms (179) and drawn with Cytoscape (v3.9.1). For every nitrogen group, differentially expressed genes induced by heat stress with Q-value < 0.05 were used for KEGG enrichment and GO enrichment analysis using phyper and TermFinder packages in R. Enrichment plots were drawn using the online tool ImageGP (175). Further detailed information about software parameter setting can be found in supporting information (Table S9).

### Statistical analysis

To test the effects of temperature and nitrogen types on repeatedly collected physiological traits, repeated measures ANOVA followed by Bonferroni *post hoc* test was conducted, combined with multivariate ANOVA for further quantifying the temperature and nutrient effects on every sampling day. To simplify the RMANOVA and MANOVA analysis for cell concentration and *Fv/Fm*, only data with 3-day intervals were chosen for statistical analysis. To test the effect of medium ammonium concentration on cell growth with 882 µM nitrate treatment as the control group, one-way repeated measures ANOVA analyses for each temperature treatment followed by MANOVA were performed. Other data were analyzed using two-way ANOVA followed by Bonferroni *post hoc* test.

For ANOVA analysis, data with studentized residuals >3 or <−3 were considered outliers and were removed before analysis. Statistical analyses were performed using IBM SPSS Statistics 25.

## Data Availability

Raw sequencing data are available at NCBI in the Sequence Read Archive (SRA) database under BioProject no. PRJNA1092428.

## SUPPLEMENTAL MATERIAL

Supplemental figures aem.00591-24-s0001.docx Fig. S1 to S7.

Supplemental tables aem.00591-24-s0002.xlsx Tables S1 to S9.

ASM does not own the copyrights to Supplemental Material that may be linked to, or accessed through, an article. The authors have granted ASM a non-exclusive, world-wide license to publish the Supplemental Material files. Please contact the corresponding author directly for reuse.
