# Supplementary material for: Nitrogen source type modulates heat stress response in coral symbiont (Cladocopium goreaui)
Source: Appl Environ Microbiol. 2025 Jan 7;91(2):e00591-24. doi: 10.1128/aem.00591-24 (PMC11837503; doi:10.1128/aem.00591-24)
Supplement: Supplemental figures — Fig. S1 to S7. [file aem.00591-24-s0001.docx]

**Supplementary figures**

**
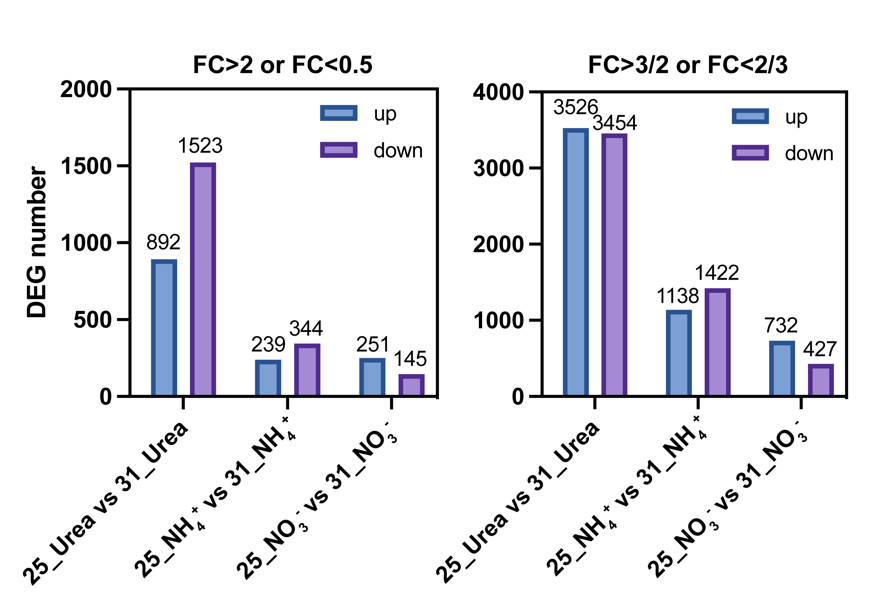
**

**Fig. S1. Number of HS-DEGs with FC cutoff values of 2 and 1.5.**


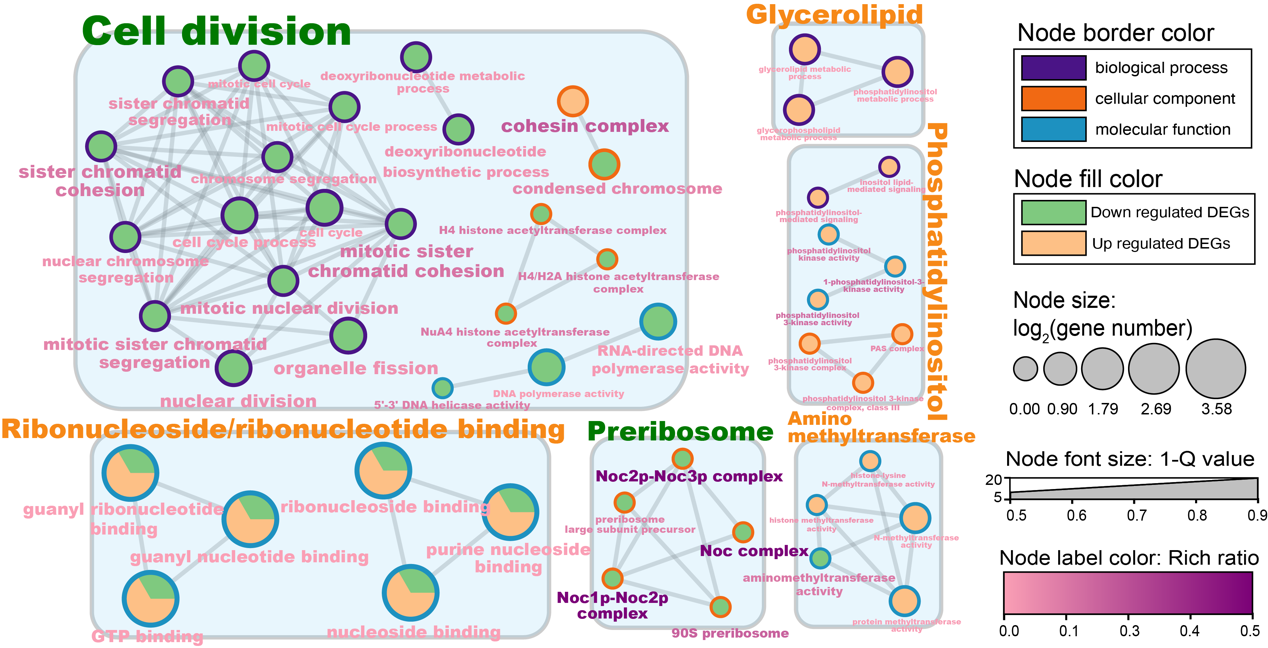


**Fig. S2. GO functional clustering chart of coHS-DEGs based on GO terms’ semantic similarities.**


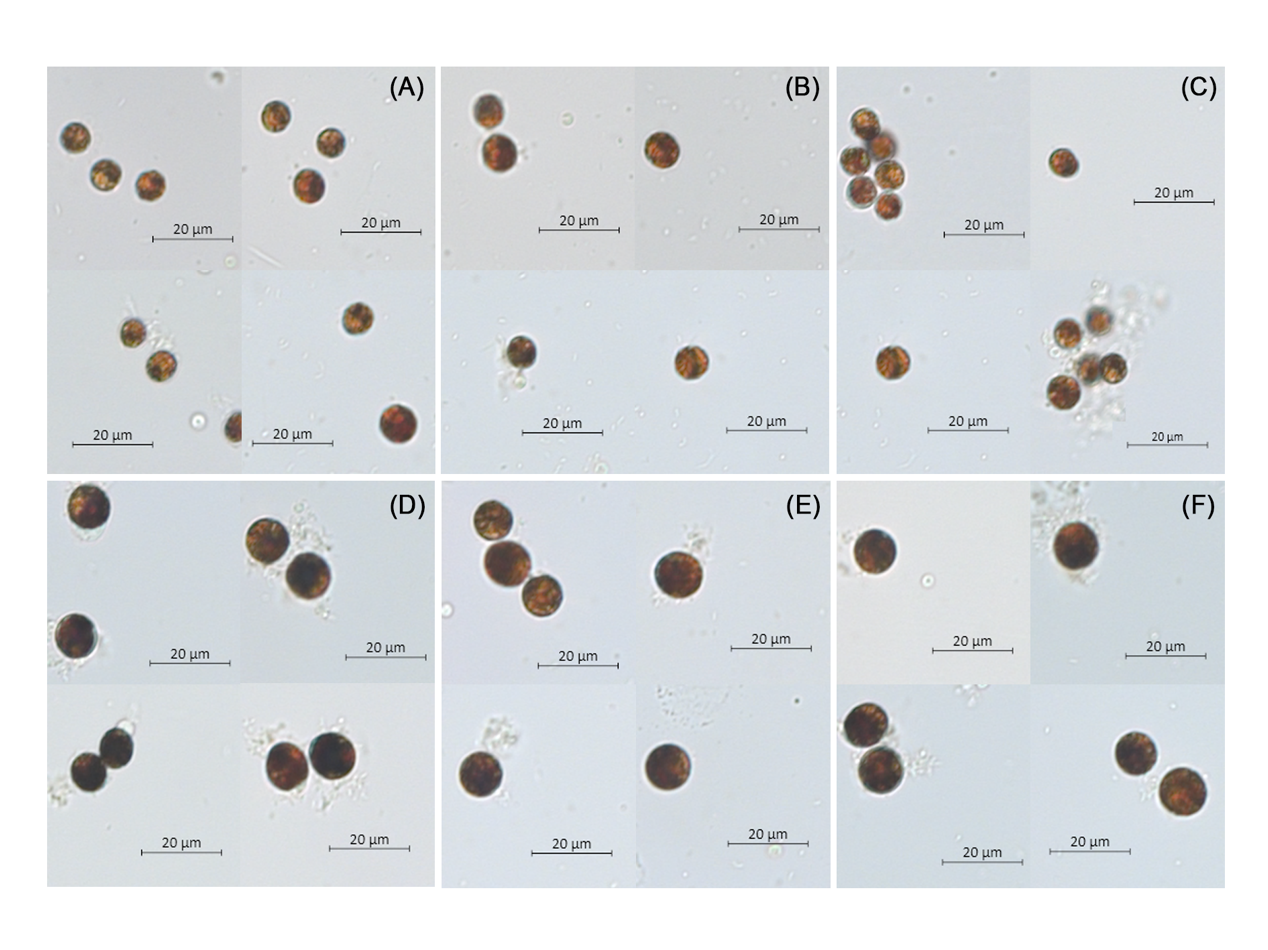


**Figure S3. Representative Lugol’s solution-stained *C. goreaui* cells collected on day 12 of the cultivation from different groups.** (A) 25˚C_ NH_4_^+^, (B) 25˚C_ NO_3_^-^, (C) 25˚C_Urea, (D) 31˚C_ NH_4_^+^, (E) 31˚C_ NO_3_^-^, (F) 31˚C_Urea.

**
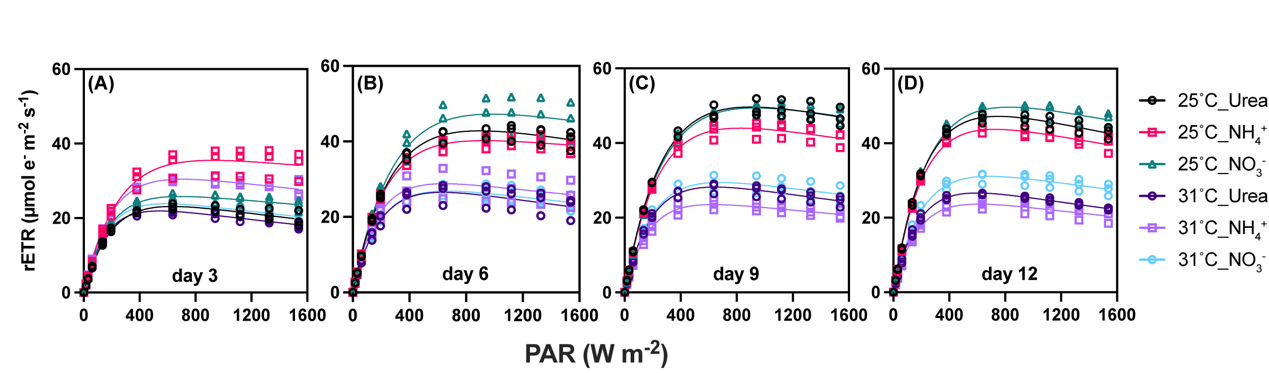
**

**Figure S4. Rapid light curves of *C. goreaui* on different culture days (A-D).**


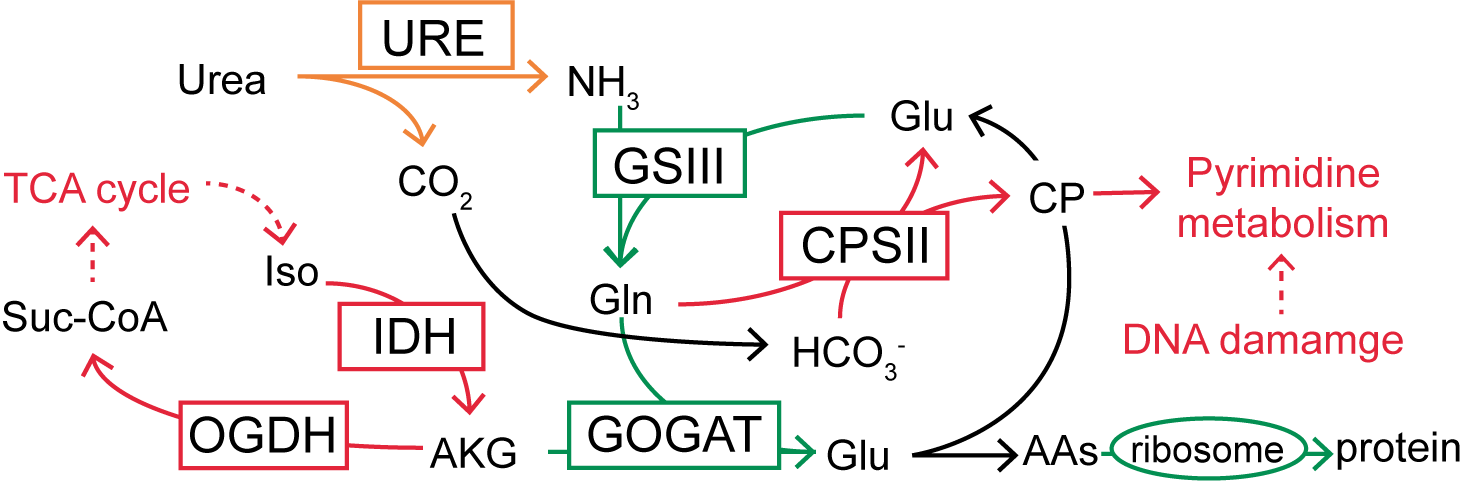


**Figure S5. Schematic of transcriptomic responses of nitrogen assimilation in *C. goreaui* cells under urea supply to heat stress.** Red, orange and green frame and arrow indicate HS-upregulated DEGs, significantly upregulated genes but with FC < 1.5, and HS-downregulated DEGs. Red fonts indicate HS-upregulated pathways. Abbreviations of metabolites: Glu: glutamate; Gln: glutamine; AAs: amino acids; AKG: α-ketoglutarate; Iso: isocitrate; Suc-CoA: succinyl-CoA; CP: carbamoyl phosphate; Arg: arginine. Information about enzyme abbreviations can be found in Table S3.

­­
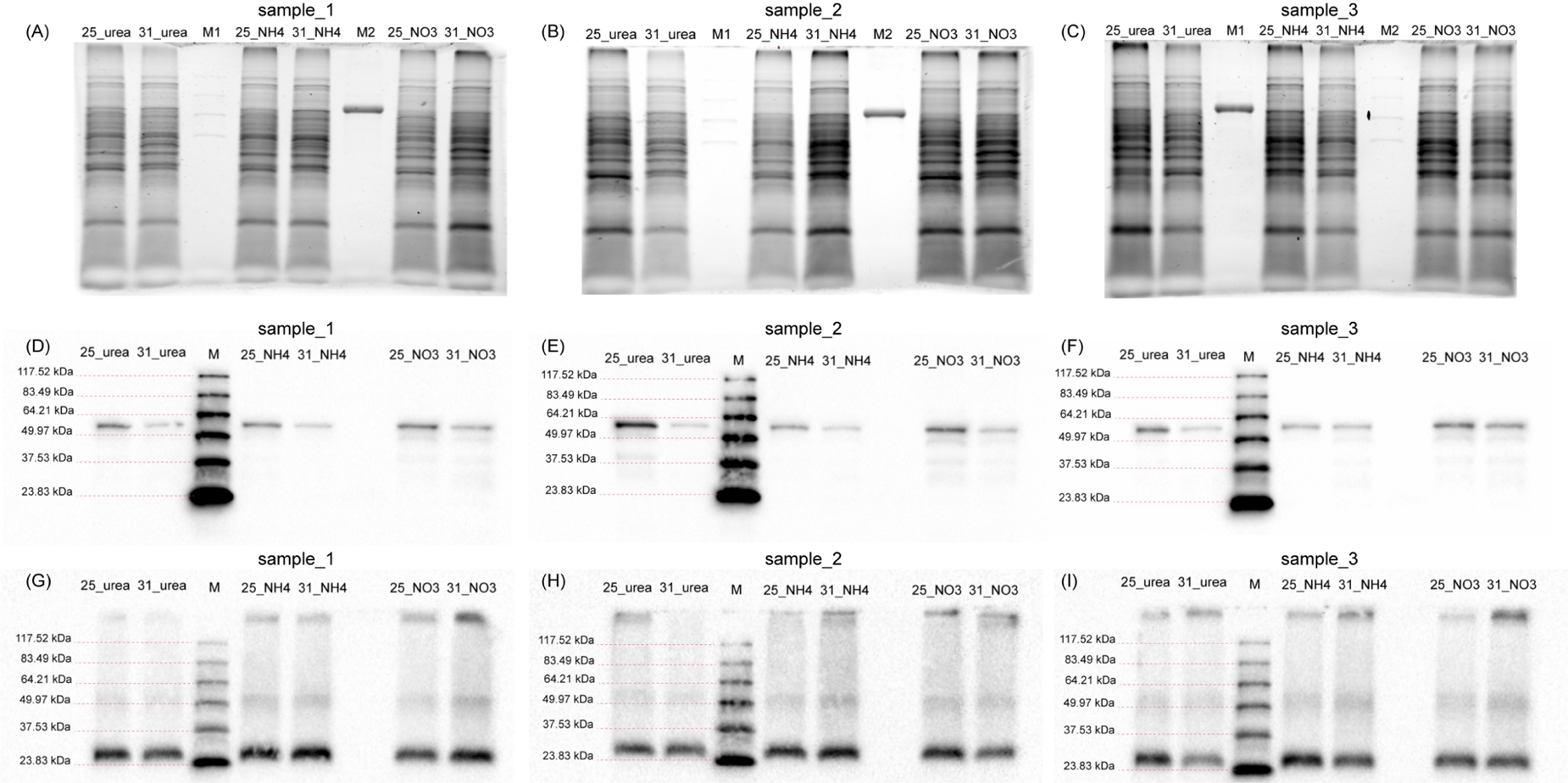


**Figure S6. Raw figures of western blotting of Rubisco and PSII D1 protein.** (A-C) Stain-free blot images of total protein. (D-F) Rubisco antiserum probed images. (G-I) anti-D1 probed images. Figures from left to right represent proteins extracted from three different replicate samples (sample_1, sample_2 and sample_3). The 3rd and 6th lanes represent molecular marker proteins. The remaining lanes showed proteins extracted from 25˚C_Urea (lane 1), 31˚C_Urea (lane 2), 25˚C_NH_4_^+^ (lane 4), 31˚C_NH_4_^+^ (lane 5), 25˚C_NO_3_^-^ (lane 7) and 31˚C_ NO_3_^-^ (lane 8).


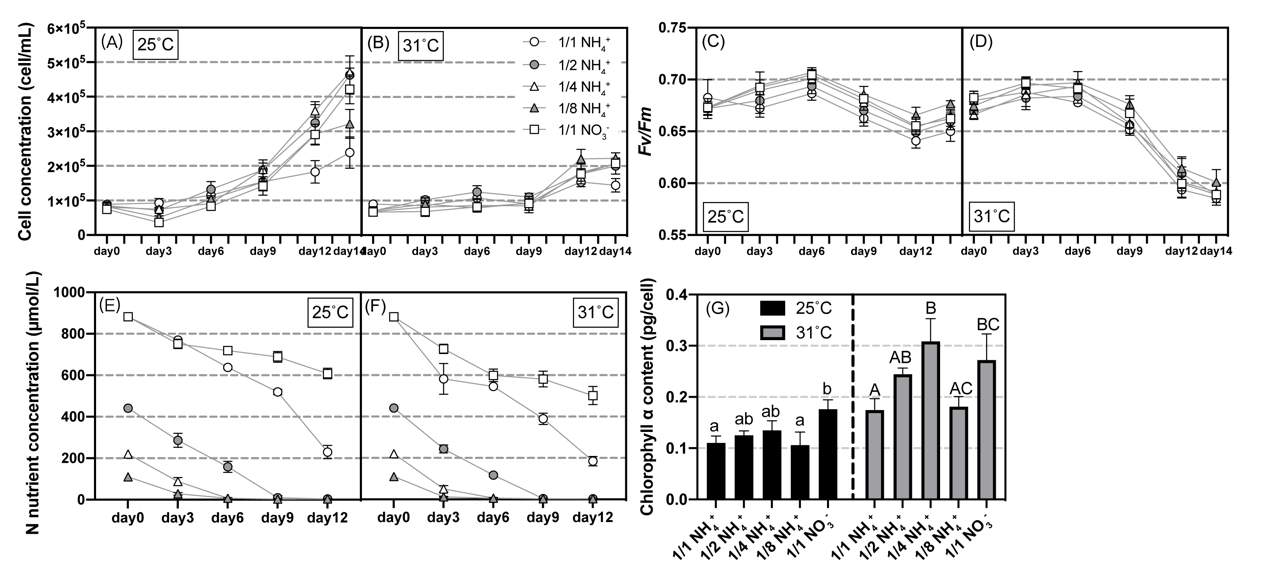


**Figure S7. Impacts of ammonium concentration on population growth, photosynthesis and nitrogen uptake of *C. goreaui* under 25 and 31 ˚C***.* (A-B) Growth curve. (C-D) *Fv/Fm*. (E-F) N nutrient depletion in culture medium. (G) cellular chlorophyll *a* contents. Data in (G) were collected on the 9th day of the cultivation. Different letters indicate significant differences among nutrient treatments under the same temperature condition. The data are mean ± SD (n=3).
